# Development of a CRISPR/Cas9 RNP-mediated genetic engineering system in *Paecilomyces variotii*

**DOI:** 10.71150/jm.2502011

**Published:** 2025-06-30

**Authors:** Hui-Gang Han, Rutuja Nandre, Hyerang Eom, Yeon-Jae Choi, Hyeon-Su Ro

**Affiliations:** Department of BioMedical Bigdata (BK21) and Research Institute of Life Sciences, Gyeongsang National University, Jinju 52828, Republic of Korea

**Keywords:** *Paecilomyces variotii*, heterologous protein, gene editing, RNP, HDR

## Abstract

A thermophilic strain of *Paecilomyces variotii* (MR1), capable of surviving temperatures above 40°C, was isolated from a paper mill and investigated as a host for heterologous protein production. To prevent environmental dissemination of spores, UV mutagenesis was employed to create a conidia-deficient strain, UM7. This strain underwent gene editing using Cas9-gRNA ribonucleoprotein (RNP) with HR donor DNA fragments, incorporating promoter sequences amplified from the genomic DNA of *P. variotii* (P_H4_, P_P2_, P_S8_, P_tub_, P_tef1_, and P_gpdA_), along with a signal sequence-tagged eGFP, flanked by 5’-upstream (336 bp) and 3’-downstream (363 bp) regions of *pyrG*. Co-transformation of HR donor DNA with RNP into protoplasts yielded 48 mutant *pyrG* transformants capable of surviving in the presence of 5-fluoroorotic acid (5-FOA). Sequence analysis identified 16 of the 48 *pyrG*-disrupted mutants carrying complete HR donor DNAs with the six different promoter sequences, indicating successful homology-directed repair (HDR). Evaluation of promoter strength revealed that P_gpdA_ was the most effective for intracellular GFP production; however, it resulted in negligible extracellular GFP signal under all promoter conditions. A newly edited strain with an HDR integration module connecting P_gpdA_ directly to eGFP, without the signal sequence, exhibited enhanced GFP expression in both mycelial cells and culture broth, suggesting that the signal peptide negatively affect protein expression and secretion. This work represents the first successful RNP-mediated gene editing in *P. variotii*, contributing to the application of this thermophilic fungus in protein production.

## Introduction

Filamentous fungi inhabit diverse ecological systems. These fungi are widely studied for industrial applications due to their ability to secrete large quantities of proteins, especially hydrolytic enzymes, into the extracellular environment (Fleißner and Dersch, 2010; Herrera Bravo de Laguna et al., 2015; Wang et al., 2020; Ward, 2012). This high secretion capacity enables the extracellular production of heterologous proteins, reducing the need for complex purification processes. Unlike prokaryotes, filamentous fungi can perform intricate post-translational modifications, such as glycosylation, protease cleavage, and disulfide bond formation. This unique capability makes them valuable in the production of medicinal proteins, as they help alleviate hyper-mannosylation, which can reduce the efficacy and half-life of heterologous proteins (Wang et al., 2020). Due to these advantages, heterologous protein expression systems in filamentous fungi have been widely studied, particularly in the *Aspergillus* and *Trichoderma* genera. For example, glucoamylase and cellulase have been successfully produced in culture broth at concentrations of 30 g/L and 100 g/L, respectively (Ward, 2012). However, heterologous protein yields in filamentous fungi are generally lower than those of native proteins due to factors such as transcription and translation efficiency, secretion pathways, pre-secretory degradation of misfolded proteins, and post-secretory degradation. Enhancing heterologous protein production efficiency requires genetic engineering approaches to optimize host strains. Among filamentous fungi, *Aspergillus* species—such as *A. niger* and *A. oryzae*—are extensively studied for genetic tool development, including promoters, transformation methodologies, and selection markers, due to their safety and high productivity in foreign gene expression (Fleißner and Dersch, 2010). These fungi are commercially employed for the large-scale production of numerous enzyme proteins, including aminopeptidase, amylase, arabinofuranosidase, asparaginase, catalase, and glucanase (Li et al., 2022; Wang et al., 2020).

CRISPR/Cas9-mediated gene editing is an emerging technology in the genetic engineering of filamentous fungi. It makes a double strand break (DSB) at a specific chromosomal site by the endonuclease activity of crRNA-guided Cas9. Disruption of gene occurs during nonhomologous end-joining (NHEJ) repair process (Critchlow and Jackson, 1998). Insertion of foreign DNA fragment through homology-directed recombination (HDR) is also facilitated by co-transformation of the gene-of-interest flanked by homologous sequences to the target site with the CRISPR/Cas9 system (Eom et al., 2025; Kim et al., 2024; Komor et al., 2017). Numerous papers have been published in gene editing of filamentous fungi and macrofungi (mushrooms) for last decade (Jiang et al., 2021; Shen et al., 2023). Delivery of Cas9 and guide RNA (gRNA) to the mycelial cells are one of the major issues. *Agrobacterium tumefaciens*-mediated transformation (ATMT) and PEG-mediated transformation (PMT) with plasmid DNA harboring Cas9-gRNA are the most frequently chosen delivery (Choi et al., 2023a; Shen et al., 2023). Direct introduction of Cas9 protein-gRNA ribonucleoprotein complex (RNP) to the cytoplasm of protoplasts has become an option to generate edited organisms which is advantageous over

ATMT and PMT since it essentially does not leave foreign DNA fragments (Boontawon et al., 2021; Choi et al., 2023b, 2025; Eom et al., 2023; Kim et al., 2024; Mout et al., 2017; Pohl et al., 2016; Vonk et al., 2019; Zou et al., 2021).

*Paecilomyces variotii* is a thermophilic fungus belonging to the order Eurotiales, found from food, soil, and indoor environments. The ascospores of *P. variotii* exhibit high thermal resistance, enduring heat treatment at 85°C for 15 min, and depending on the strain, it can grow as vegetative mycelia at 50°C (Houbraken et al., 2006). It can decompose lignocellulosic biomass, like corn cobs, wheat bran, and sugarcane bagasse, which are difficult for typical microbes to degrade, enabling their utilization in ethanol production (Zerva et al., 2014) and aromatic pollutants as well (Wang et al., 2010). It has been employed for the production of microbial protein as animal feed (e Silva et al., 1995; Giannoutsou et al., 2012), thermostable enzymes such as tannase, amylase, phytase, pectinase, and xylanase (Abdella et al., 2021; Battestin and Macedo, 2007; Michelin et al., 2010; Patil et al., 2012; Schons et al., 2012). The first successful gene editing was reported to create a melanin-deficient strain by using ATMT (Urquhart et al., 2018) with plasmid-borne CRISPR/Cas9 targeting the kusA gene (Seekles et al., 2021).

We recently isolated a strain of *P. variotii* from a hot-pressing facility in a paper mill, which exhibited heat resistance up to 50°C and the ability to degrade lignocellulosic pulp Given these traits, we sought to develop this strain as a host for heterologous protein production at elevated temperatures. To achieve this, we developed a heterologous gene expression system using Cas9-gRNA RNP-mediated gene editing. We introduced a GFP-encoding gene under the control of six strong promoter sequences, flanked by homologous regions of the target site, *pyrG*. As a result, we successfully performed RNP-mediated gene editing in *P. variotii* for the first time, demonstrating efficient gene replacement via HDR.

## Materials and Methods

### Fungal strains and culture conditions

*Paecilomyces variotii* MR1 was isolated from a fungi-contaminated pulp sample obtained from a paper factory in southern Korea. MR1 and all derived *P. variotii* strains were maintained on potato dextrose agar (PDA). The *pyrG* deletion mutant strain was screened on minimal media [MMS: Czapek dox broth (33.4 g/L), 1 M sucrose, uracil (0.02 g/L), uridine (5 g/L), and agar (1.2%)] supplemented with 2 g/L 5-fluoroorotic acid (5-FOA).

### Generation of sporeless mutant strain

After culturing the MR1 strain on PDA at 40°C for 7 days, conidia were collected in a saline solution (0.9% NaCl, 0.02% Tween 80). The conidial suspension (10^7^ spores/ml) was spread onto PDA and then exposed to UV light (254 nm) for 1 min at a distance of 30 cm. Colonies that did not produce spores, thus forming white colonies after culturing for 2 days at 40°C, were isolated from the plate. The isolated mycelial cells were cultured on PDA for 5 days at 40°C. The spores were collected using 1-ml of the saline solution. The spore concentration was determined using a hemocytometer under an optical microscope.

### Preparation of Cas9 protein and gRNA

The Cas9 protein was prepared using *Escherichia coli* BL21 strain harboring the pTrc-Cas9 vector as previously described (Eom et al., 2023). The expressed Cas9 protein was purified through Ni-NTA column chromatography, and the eluted protein was dialyzed against a dialysis buffer [20 mM HEPES-KOH, 500 mM KCl, 10% glycerol, 1 mM EDTA, and 1 mM DTT (pH 7.5)].

The crRNA targeting the *pyrG* gene of *P. variotii* (GenBank ID: XP_028486184) was designed using Chopchop (https://chopchop.cbu.uib.no/). The *pyrG* encodes orotidine 5-phosphate decarboxylase which converts 5-fluoroorotic acid (5-FOA) into lethal 5-fluorouracil (5-FU). Disruption of *pyrG* enables positive selection on 5-FOA whereas the wild-type fails to survive due to the production of 5-FU (Eom et al., 2023). Using the pHAtC-AbCas9/gRNA vector (Choi et al., 2023a) as a template, a gRNA backbone DNA containing T7 promoter (P_T7_), crRNA, and tracrRNA was amplified with primer set 1 (Table. 1 and Fig. S1). The resulting DNA fragment was employed to synthesize gRNA via an in vitro transcription using RNA synthesis kit (NEB, USA) and subsequently purified by an RNA purification kit (Invitrogen, USA).

### Promoter sequence acquisition

Promoter sequences known for their high-level expression of downstream genes in filamentous fungi, such as histone H4 (P_H4_) (Rendsvig et al., 2019), 60S acidic ribosomal protein P2 (P_P2_) (Khang et al., 2010), 40S ribosomal protein S8 (P_S8_) (Mózsik et al., 2019), beta tubulin (P_tub_) (Matsu-Ura et al., 2015), TEF1 alpha (P_tef1_) (Kitamoto et al., 1998), and glyceraldehyde-3-phosphate dehydrogenase (P_gpdA_) (Punt et al., 1990), were sourced from the genomic sequence of *P. variotii* available in Mycocosm (https://mycocosm.jgi.doe.gov/Paevar1/Paevar1.home.html).

### Generation of HR donor DNA fragments

The HR donor DNA containing the promoter sequence followed by the reporter gene (eGFP), flanked by the upstream 336-bp region (HR-L) and the downstream 363-bp region (HR-R) of *pyrG*, was generated following the process outlined in Fig. S2. The primer sets utilized in this process are provided in Table S1. The HR-L and HR-R regions were amplified from the chromosomal DNA of *P. variotii* UM7 and assembled into the pTOP plasmid (Enzynomics, Korea) along with internal multiple cloning site (MCS) from pRS425, resulting in the creation of pUP. Subsequently, the eGFP gene, featuring the signal sequence from *P. variotii* tannase (GenBank ID: XP_028486386), was amplified and integrated into NotI site of the MCS region to produce pUPG. The promoter sequence, amplified from the chromosomal DNA of *P. variotii* UM7, was inserted into the SalI/SpeI sites of the MCS site at pUPG, resulting in the generation of pUPG-PP_H4_~P_gpdA_. The plasmid pUPG-P_gpdA_-SP was constructed using the P_gpdA_ and eGFP DNA fragments amplified by primer sets 13 and 14 (Table S1). The HR donor DNA fragments were amplified from pUPG-PP_H4_~P_gpdA_ and pUPG- P_gpdA_-SP using the primer set 15 (Table S1). Signal sequence in the tannase sequence was predicted by SignalP 6.0 (https://services.healthtech.dtu.dk/services/SignalP-6.0/). The tannase signal peptide was selected due to *P. variotii*'s documented ability to efficiently secrete a substantial quantity of tannase into the culture broth (Patil et al., 2012; Schons et al., 2012).

### Protoplast generation and Cas9-gRNA RNP mediated gene editing

The protoplast was generated by using a previously described method with slight modification (Seekles et al., 2021). In brief, UM7 was cultured in 100 ml of PDB for 17 h at 30°C with agitation (170 rpm). The mycelia obtained through filtration with miracloth were washed twice with SMC buffer (1.33 M D-sorbitol, 5.5 g/L CaCl_2_, and 20 mM MES, pH 5.8) and then treated with a solution of cell wall lysing enzymes [200 mg lysing enzyme (Sigma-Aldrich, USA)/10 ml SMC] at 37°C for 3 h with gentle shaking. The reaction mixture was filtered through miracloth, and the filtrate was subsequently centrifuged at 2,000 × g for 10 min at 4°C. The collected protoplasts were suspended in STC buffer (1.33 M D-sorbitol in TC buffer) and diluted to a concentration of 10^7^ protoplasts/ml STC buffer. TC buffer was composed of CaCl_2_ (5.5 g/L) and 10 mM Tris base (pH 5.8).

PMT was conducted following a previously reported methodology with slight modification (Moon et al., 2021). Briefly, 200 μl of the protoplast solution was mixed with 10 μl of Cas9-gRNA RNP solution, containing 10 μg of Cas9 protein and 5 μg of gRNA, along with 10 μl of HR donor DNA solution (3 μg), 0.006% Triton X-100, and 50 μl PTC buffer [2.5 g of polyethylene glycol 6000 (PEG)/10 ml TC buffer] then incubated for 20 min on ice. Subsequently, the solution was mixed with 3 ml of PTC buffer and incubated for an additional 50 min at room temperature. The resulting solution was mixed with 6 ml of STC buffer. The protoplast mixture solution was spread on a 5-FOA MMS agar plate and incubated for 6–9 days at 30°C. The mycelial colonies grown from the medium were transferred to a new 5-FOA MMS agar for further analysis. Successful gene editing in the transformants was confirmed by sequence analysis of the *pyrG* region, amplified by the primer sets 1 and 15 (Table S1).

### Fluorescence microscopy and spectral analysis

The intracellular GFP was analyzed using a fluorescence microscope (Evos FL Auto 2, Thermo Fisher, USA). For the GFP secreted to culture broth, the mycelia were cultured in MMS at 40°C for 5 days with agitation (170 rpm). The culture broth was subjected to fluorescence analysis using luminescence spectrometer (LS 55, PerkinElmer, USA). The emission spectrum was acquired after excitation at 510 nm. Fluorescence intensity of GFP inside cells was evaluated by the density of GFP signal analyzed by ImageJ software (https://imagej.net/).

### Statistical analysis

Three independent measurements were conducted to perform statistical analysis. The statistical significance of the differences in the spore number and the mycelial growth were tested by one-way ANOVA followed by Tukey's Honest Significant Difference (HSD) test at an alpha level of 0.01.

## Results

### Generation of sporeless strain

**Fig. 1.**
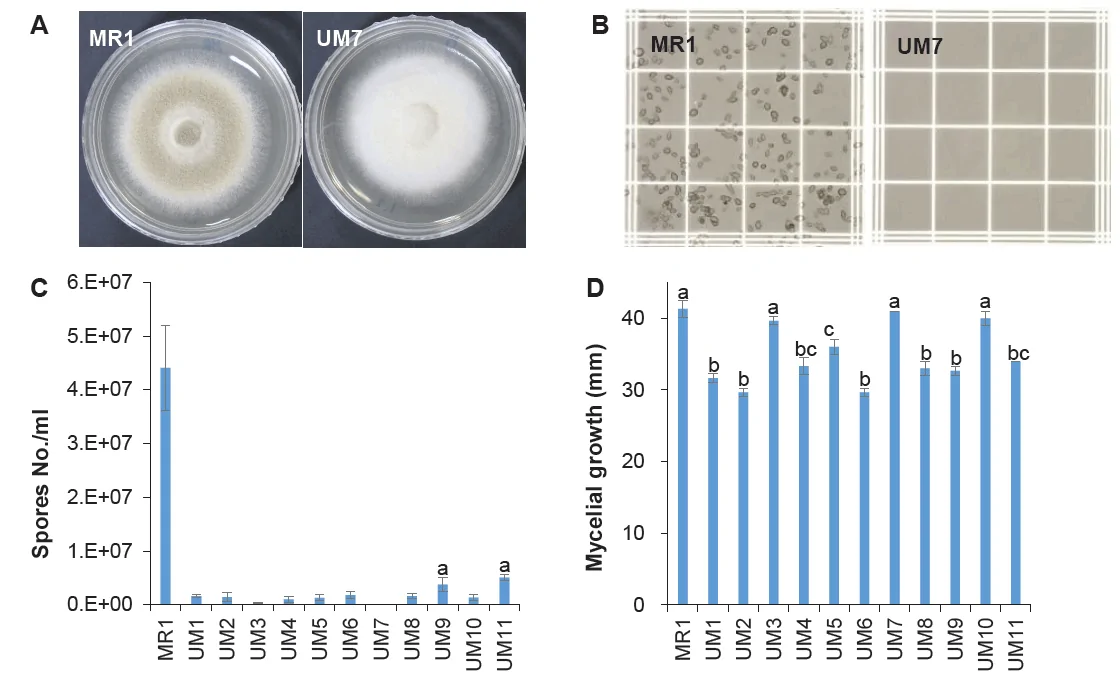
Generation of sporeless strain of *P. variotii*. Conidia spread on PDA were exposed to UV light for 1 min. The grown mycelial colonies showing white color were isolated to for spore counting. (A) Morphology of the wild-type (MR1) and the sporeless mutant (UM7). (B) Spore counting on hemacytometer. (C) Number of spores in different mutant strains. (D) Mycelial growth characteristics of the mutant strains on PDA for 3 days. Three independent measurements were conducted to perform statistical analysis. The statistical significance of the differences in the spore number and the mycelial growth were tested by one-way ANOVA followed by Tukey's Honest Significant Difference (HSD) test at an alpha level of 0.01.

Spore dissemination is the primary mechanism of fungal dispersion. To prevent environmental contamination by genetically modified mutants, UV mutagenesis was performed to generate a sporeless strain of *P. variotii*. UV irradiation of germinating spores resulted in 11 mutants exhibiting white mycelia, indicating a loss of sporulation (Fig. 1A). Among these, UM7 was identified through microscopic spore counting as entirely incapable of spore production (Fig. 1B and 1C). In a comparative growth assay, UM7 displayed a growth rate comparable to its wild-type MR1 strain (Fig. 1D). The genetic stability of UM7 was further confirmed through more than 10 consecutive transfers on fresh PDA, following 5 days of growth at 40°C.

### Integration of HR donor DNAs with various promoters using Cas9-gRNA ribonucleoprotein complex

**Fig. 2.**
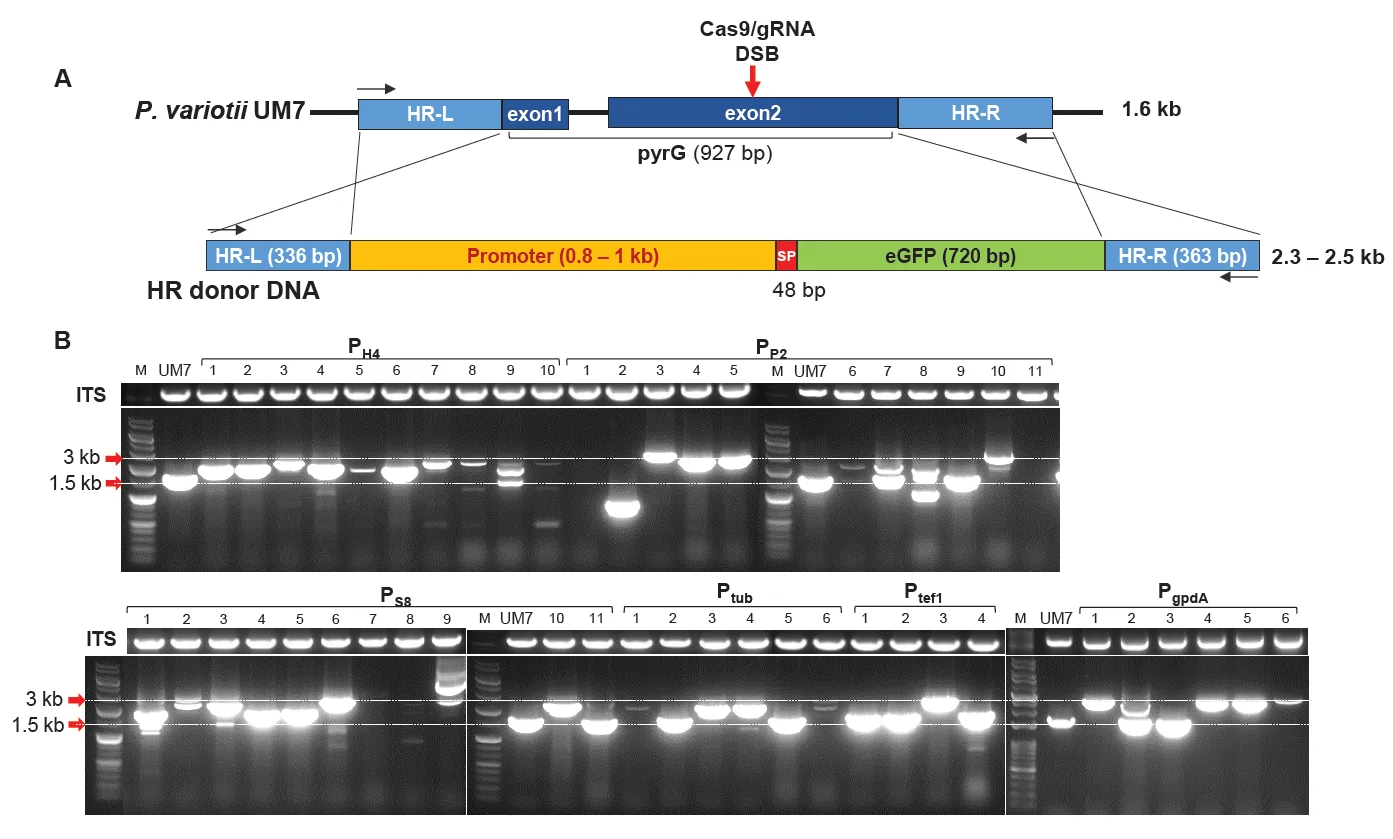
Cas9-gRNA ribonucleoprotein-mediated gene editing of *P. variotii* UM7. (A) Schematic representation of homology-directed recombination (HDR). The HR donor DNA was designed to replace the *pyrG* ORF during the repair of a double-strand break (DSB) induced by Cas9-gRNA ribonucleoprotein. The DSB site is indicated by a red arrow. The primer binding sites used for PCR analysis are shown as black arrows. (B) PCR analysis of transformants amplified using primers targeting the HR-L and HR-R regions. Transformants carrying six different promoters (P_H4_, P_P2_, P_S8_, P_tub_, P_tef1_, and P_gpdA_) that survived on 5-FOA selection were isolated and subjected to colony PCR. Internal transcribed spacer (ITS) sequences were concomitantly amplified under the same PCR conditions as control experiments.

To investigate whether *P. variotii* UM7 could be used for the heterologous protein expression, we cloned eGFP expression modules containing six types of constitutive promoters from *P. variotii*, including P_H4_, P_P2_, P_S8_, P_tub_, P_tef1_, and P_gpdA_ (Fig. S2). These modules were used as HR donor DNA fragments to perform PEG-mediated transformation, allowing HDR to substitute *pyrG* during DSB repair caused by Cas9-gRNA RNP (Fig. 2A). As a result, 48 *pyrG* transformants capable of surviving in 0.2% 5-FOA were obtained. Their *pyrG* loci were PCR-amplified to analyze gene editing patterns at the target site. Agarose gel electrophoresis of the amplified products revealed that most amplicons from the transformants were larger than the expected 1.6 kb size of the wild-type *pyrG* in UM7 (Fig. 2B). However, some amplicons, such as P_P2_-9, P_S8_-11, P_tub_-2, and P_tub_-5, were approximately the same size as the wild-type *pyrG*. Notably, the amplicon P_P2_-2 produced the smallest DNA band, measuring only 0.9 kb. Additionally, transformants such as P_H4_-3, P_H4_-7, and P_P2_-4 displayed PCR band sizes consistent with the expected insertion of HR donor DNA. In contrast, strains like P_P2_-1, P_P2_-11, and P_S8_-7 failed to amplify the *pyrG* locus, despite successful amplification of the internal transcribed spacer (ITS) sequence, which includes ITS1, 5.8S, and ITS2 located between 18S and 28S rDNA. This suggests the presence of large insertions or deletions extending beyond the target *pyrG* sequence (Fig. 2B).

### Sequence analysis of the transformants

**Fig. 3.**
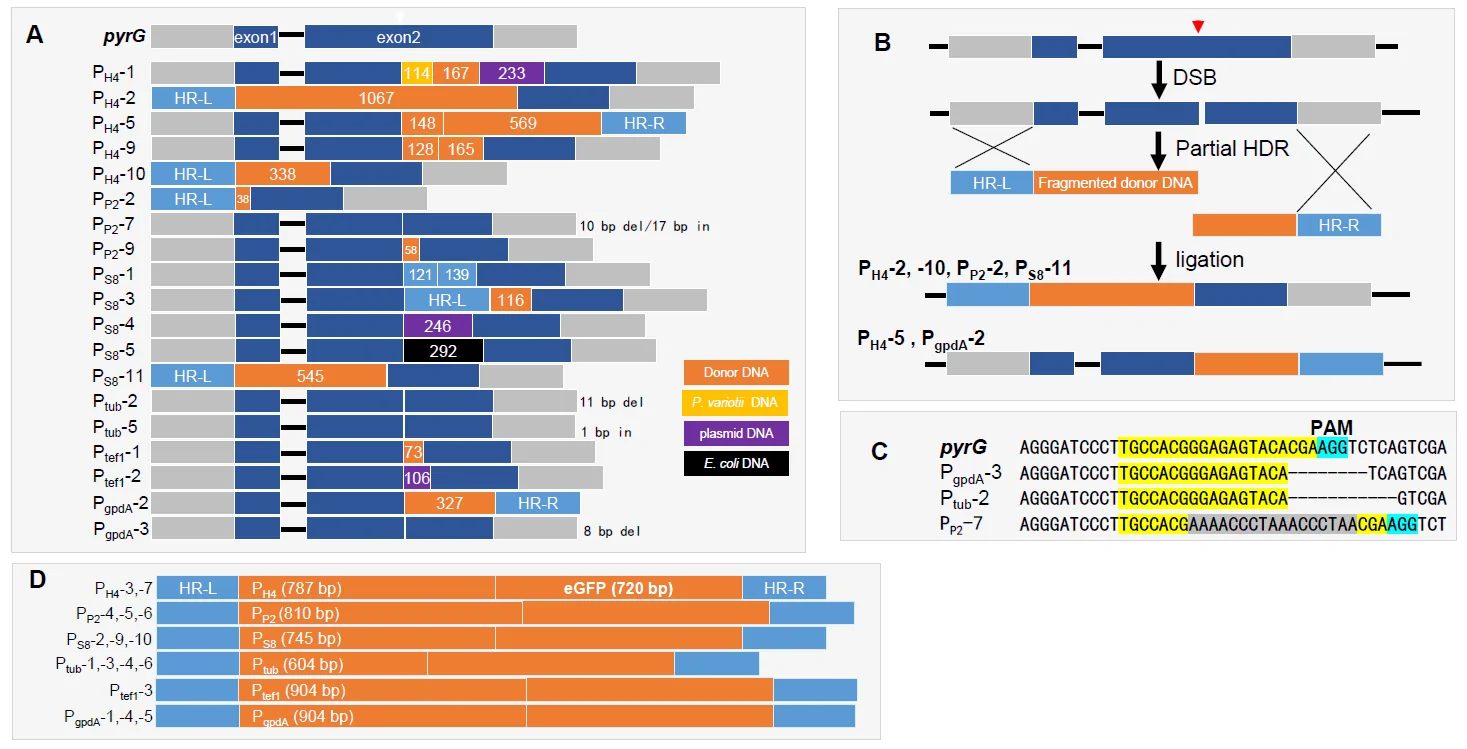
Sequence analysis of gene edited transformants. (A) Representative sequences generated by NHEJ and partial homology-directed repair (HDR). Inserted sequences are color-coded: orange for HR donor DNA, yellow for host chromosomal DNA, purple for plasmid DNA, and black for *E. coli* chromosomal DNA. (B) Schematic representation of the generation of partial HDR/NHEJ transformants. Fragmented donor DNAs containing either HR-L or HR-R underwent HDR followed by ligation. Transformants P_H4_-2, P_H4_-10, P_P2_-2, P_S8_-3, and P_S8_-11 were generated via HDR with HR-L-fragmented donor DNA, while P_gpdA_-2 was generated via HDR with fragmented donor DNA containing HR-R. (C) Two transformants exhibited deletions around the DSB site. The RNP recognition sequences are highlighted in yellow. (D) The transformants generated by complete HDR.

DNA sequences of the 48 PCR amplicons in Fig. 2B were analyzed. As a result, 30 transformants were found to contain inserted DNA fragments of varying lengths, ranging from 1 bp to over 1 kb (Fig. 3 and Data S1). Sequence analysis of the inserted DNA revealed that most were fragmented HR donor DNAs, as observed in P_H4_-2, P_H4_-5, P_H4_-9, P_H4_-10, P_P2_-2, P_P2_-9, P_S8_-1, P_S8_-3, P_S8_-11, P_tef1_-1, and P_gpdA_-2 (Fig. 3A and Data S1). In contrast, P_S8_-4 and P_tef1_-2 contained fragmented plasmid DNA, while P_S8_-5 carried fragmented *E. coli* chromosomal DNA. Notably, P_H4_-1 carried three pieces of fragmented DNAs, consisting of host chromosomal DNA (114 bp), donor DNA (167 bp), and plasmid DNA (233 bp) (Fig. 3A). The insertion of various fragmented DNAs was also observed in our previous RNP-mediated gene editing study with *Ganoderma lucidum* (Eom et al., 2023). Partial HDR, followed by NHEJ repair of broken HR donor DNA, was detected in P_H4_-2, P_H4_-5, P_H4_-10, P_P2_-2, P_S8_-11, and P_gpdA_-2 (Fig. 3B).

In filamentous fungi, RNP-mediated gene editing predominantly results in deletions between the third and fourth bases upstream of the PAM sequence (Eom et al., 2023). However, our gene editing data indicate that deletions occurred in only two transformants, where P_gpdA_-3 and P_tub_-2 exhibited deletions of 8 bp and 11 bp downstream of the DSB site, respectively (Fig. 3C). P_P2_-7 contained a DNA fragment of 17-bp after 10-bp deletion of the Cas9 recognition sequence (Fig. 3C). Sixteen out of 48 transformants (33.3%) carried complete HR donor DNAs, indicating successful HDR at the Cas9-gRNA RNP-induced DSB site (Fig. 3D and Data S1). These complete HDR transformants included two P_H4_-GFP (P_H4_-3 and -7), three P_P2_-GFP (P_P2_-4, -5, and -6), three P_S8_-GFP (P_S8_-2, -9, and -10), four P_tub_-GFP (P_tub_-1, -3, -4, and -6), one P_tef1_-GFP (P_tef1_-3), and P_gpdA_-GFP (P_gpdA_-1, -4, and -5).

The observed HDR rate was exceptionally high, as DSB repair in filamentous fungi predominantly occurs through NHEJ (Critchlow and Jackson, 1998).

### Expression of GFP

**Fig. 4.**
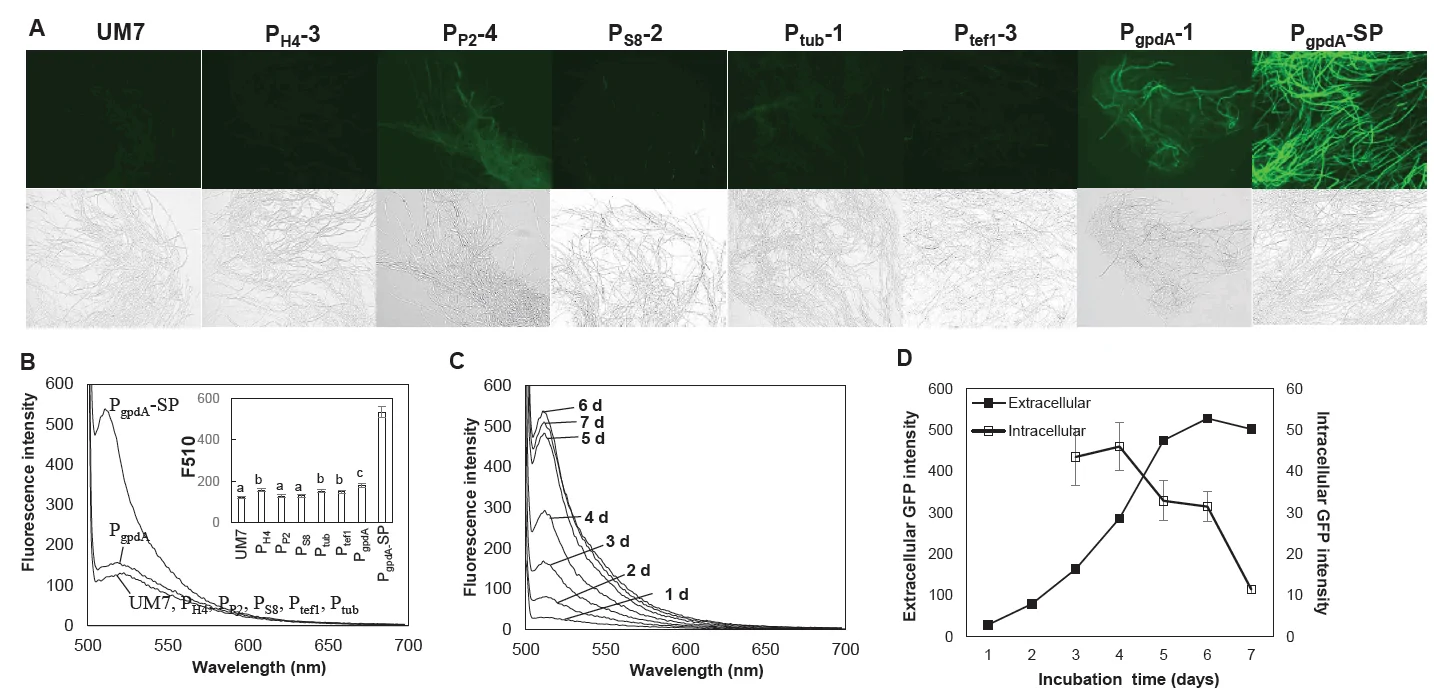
Expression of GFP in the HDR mutants with different promoters. (A) Fluorescence microscopic observation of the mycelia of the HDR mutants. P_gpdA_-SP is the transformant without signal peptide. (B) Extracellular GFP production in the culture broth. The statistical analysis on the fluorescence intensity at 510 nm (F510) are shown in the inset figure. (C) Time-dependent production of extracellular GFP from the P_gpdA_-SP transformant. (D) Expression of GFP in the mycelial cells (Intracellular) and to the culture broth (Extracellular) of the strain P_gpdA_-SP strain. The extracellular GFP fluorescence was determined bsased on the fluorescence intensity at 510 nm shown in panel (C). Intracellular fluorescence was assessed by measuring the GFP signal intensity observed under a fluorescence microscope. The fluorescence images were analyzed using ImageJ software. Error bars represent the standard errors calculated after three independent measurements.

We assessed the impact of six promoters on the expression of GFP in strains edited with RNP, each carrying distinct promoter-GFP HDR units. P_gpdA_ emerged as the most effective promoter for GFP expression among the six although, the GFP fluorescence was not prominent enough as anticipated (Fig. 4A). Remarkably, there was virtually no detectable extracellular production of GFP in the culture broth for strains featuring any of the six promoters (Fig. 4B). We hypothesized that this might be linked to the functionality of the tannase signal peptide fused to the N-terminus of GFP, as previous studies have indicated that signal peptides can influence protein expression and secretion (Liu et al., 2023; Madhavan and Sukumaran, 2014; Xu et al., 2018). To investigate this further, we engineered new RNP-driven edited strains with an HDR integration module, directly connecting P_gpdA_ to GFP to create a P_gpdA_ controlled GFP expressing strain without signal peptide (P_gpdA_-SP). The resulting P_gpdA_-SP strain exhibited enhanced GFP expression in both mycelial cells and the culture broth, as illustrated in Fig. 4A (P_gpdA_-SP) and Fig. 4B. GFP production in the culture broth continued to increase up to 6 days of cultivation, while intracellular GFP levels decreased after 4 days of incubation (Fig. 4C and 4D).

## Discussion

Unlike unicellular yeasts, genetic modification of filamentous fungi encounters significant challenges, primarily attributed to the scarcity of molecular biological tools and the difficulty in inducing homologous recombination with foreign DNA. However, the recent advent of gene editing technology has substantially eased the process of genetic modification in filamentous fungi (Jiang et al., 2021; Shen et al., 2023; Zou et al., 2021). These technological advancements are particularly pronounced in industrially crucial ascomycetes like *Aspergillus* and *Trichoderma* (Jiang et al., 2021; Shen et al., 2023), with numerous promising outcomes emerging in basidiomycetes, where genetic engineering modification was previously limited to a few species (Boontawon et al., 2021; Choi et al., 2023a; Eom et al., 2023; Vonk et al., 2019).

The primary hurdle in gene editing filamentous fungi lies in modifying target genes without leaving traces, a feat achieved through the transient expression of Cas9 and gRNA from plasmid DNA (Jiang et al., 2021; Shen et al., 2023) or the direct introduction of preassembled Cas9-gRNA RNP into the cytoplasm (Boontawon et al., 2021; Eom et al., 2023; Zou et al., 2021). Transient expression via plasmid offers advantages in transformant selection but may face challenges in achieving a sufficient expression of Cas9-gRNA due to its temporary retention. Additionally, addressing heterokaryosis observed in *A. bisporus* poses significant difficulties (Choi et al., 2023a). Conversely, RNP can circumvent the issue of Cas9-gRNA expression by introducing a sufficient amount into the cytoplasm but may encounter challenges in screening the transformant if the target editing does not produce observable phenotypes. Nevertheless, this issue can be resolved through the pooling of transformant DNAs and deep sequencing using NGS technology (Kim et al., 2020).

This study aimed to create a strain of *P. varietii*, isolated from a paper factory, to function as a heat-resistant host for expressing heterologous proteins. This pursuit stemmed from the existing constraints of available hosts, such as *Trichoderma* and *Aspergillus*, which typically thrive at temperatures below 30°C. To achieve this goal, we employed UV mutagenesis to create a sporeless strain, UM7, mitigating the risk of genetic pollution in the ecosystem that could result from the dissemination of genetically modified spores. Subsequent gene editing using Cas9-gRNA RNP targeted the *pyrG* gene of UM7 with HR donor DNA, resulting in 48 transformants. Among these, 16 were HDR transformants, while 32 were in/del mutants generated by NHEJ and/or partial HDR. Notably, the HDR rate in this study surpassed previous results with *T. reesei* (Hao and Su, 2019) and was comparable to the optimized RNP protocol supplemented with chemical reagents, such as inositol and benomyl (Zou et al., 2021). This increased HDR efficiency may be influenced by the incubation time of the protoplasts with RNP and HR donor DNA. A recent study also reported enhanced HR efficiency in *T. reesei* by extending the processing time in the PEG buffer (Zou et al., 2021).

GFP signal observed from the edited strains replacing *pyrG* ORF with six different promoter-GFP HDR units showed different amounts of the produced GFP, revealing P_gpdA_ as the best promoter. The gpdA promoter has been the most frequently employed promoter in the gene editing of filamentous fungi, including *Cordyceps militaris* (Chen et al., 2018, 2022; Meng et al., 2022), *Acremonium chrysogenum* (Chen et al., 2020), *G. lucidum* (Liu et al., 2020; Qin et al., 2017). However, we were not able to detect secreted GPF protein from the culture broth, implying troubles in the secretion through the tannase signal peptide, as evidenced by elevated GFP production by the removal of the signal peptide in front of GFP. Signal peptide is known to affect the heterologous protein production in fungi (Liu et al., 2023; Madhavan and Sukumaran, 2014; Xu et al., 2018).

In conclusion, our study successfully applied CRISPR/Cas9-mediated gene editing with HDR to address obstacles in genetically manipulating *P. variotii* for heterologous protein production. This research represents a pioneering use of RNP-mediated gene editing in *P. variotii*, contributing significantly to the improvement of protein production in the species and illuminating potential applications in the field of biotechnology.

## Data Availability

No data was used for the research described in the article.

## Supplementary Information

Fig. S1. Generation of gDNA. The DNA fragment consisted of T7 promoter-crRNA-tracrRNA was PCR-amplified using the primer set 1 (Pv pyrG gRNA1 F and Pv pyrG gRNA1 R). The resulting PCR products were subjected to in vitro transcription to produce gRNA.

Fig. S2. Construction of HR donor DNA fragments. The HR-L and HR-R regions of *pyrG*, separated by multicloning site (MCS) were inserted to the pTOP vector to make pUP. The eGFP having a tannase signal sequence was amplified and inserted into the *Not*I site of pUP to generate pUPG. The six constitutive promoter sequences were amplified from the chromosomal DNA of *P. variotii* and were inserted into the *Sal*I and *Spe*I sites of pUPG to make pUPG-P. The HR donor DNA were generated by *Kpn*I/*Apa*I digestion of the pUPG-P plasmids.

Table S1. Primers used in this study

Data S1. Sequence analysis of the transformants
