# Supplementary material for: Development of a CRISPR/Cas9 RNP-mediated genetic engineering system in Paecilomyces variotii
Source: J Microbiol. 2025 Jun 30;63(6):e2502011. doi: 10.71150/jm.2502011 (PMC13577017; doi:10.71150/jm.2502011)
Supplement: Supplementary file 1 [file jm-2502011-Supplementary-Data-S1.pdf]

Data S1. Sequence analysis of the transformants

Donor DNA (GFP) *P. variotii* chromosomal DNA Cas9 expression vector debris *E. coli* chromosomal DNA

>P_{H4}-1, 114/167/233 bp insertion

CCCTCCAAATTTACCCCTCTATCTGCATTGAACGACCATGTCTTCCAGATCGCAATTGAGATACTCTGAACGAGCACGAAACCATCCTAATGTGCTCGCG
AAGAAGCTGTTCGAAATAGCGGAGGCAAAGAAGACTAATGTCACTGTATCTGCCGATGTCACGACTACGAAGGAACTGCTGGATCTAGCTAACCGTAAGT
TGGAACTTATTACTCTCAATTACACGCATCCGCGCATTCGAAGGTCAGGTTTTTTTTAAGCTAACAATCTATCTGCCCTATACAGGTCTCGGCCCCCAT
ATCGCCGTGATCAAAACCCACATTGACATCCTATCCGACTTCAGCGAAGAGACCATCTCCGGCCTGACGGCGCTCGCGAAGAAGCACAACTTCCTCATCT
TTGAGGACCGCAAGTTCATCGATATCGGTAACACCGTCCAGAAGCAGTACCATGGTGGAGCTCTCCGGATATCTGAGTGGTCCCACATTATCAATTGCGC
CATCCTCCCCGGTGAGGGTATCGTCGAGGCTCTCGCCCAAACTGCTTCGTCGCCAGATTTCCCATACGGAAAGGAGAGGGGACTGCTGATTCTAGCAGAG
ATGACCTCCAAGGGATCCCTTGCCACGGGAGAGTACAGCTCTTCGTGAAGTCTCCGCTGCTAGAGAAGAAGTTCCTGGTCGTCGTGGTTACCCCGGTTAT
ATGTACACTGACTTGTCAACTATCTACGAACGTGCCGGCCGTGTCGAGGGCATTGAACGACCCCTCGAGGTCGACAGATGAAGGCGTTGATGGAGAAAGA
ATAGAAGCTACTTACGGCCAGTCATGTTGATAGATGTGGATTAAGATTTTTAAAGTTGTAGAGACGTGGAGATGGATGAAGTTGAAACTCGTGGATAGAG
ACTTAGCTGGATGGAGAGGCCCCCATACCTCCCCTTGTTAAAACAATAAAAAACAACTTCCACGCTCAATAAATTCTCCCAAAGAGGTTCGTTTCCCAGT
AGCTGGTTATCATTCGGATTACCGTGGCCCTCCTTTAAGCGGAGTAAAATGCTAACAGAGATCTAGGATACTAAAAAAAAATAAAAAAAGTTAGAAATCA
GAAAAGAAAACAGCATTTATAATTTCAGAAAATTCCAGACTTACAAACGGTCGAAGGTCTCAGTCGACTATGCCAGGAAGTACAAGGATTTCGTCATGGG
CTTTGTTTCGACGAGGGCATTGTCGGAGATCAAATCGGACGTGACCACGGCTTCGGATGATGAGGACTTTGTGGTTTTCACCACCGGTGTCAATCTGTCG
TCGAAGGGTGATAAGCTTGGACAGCAGTACCAGACACCTCAGTCAGCGATCGGCCGCGGTGCGGACTTTATTATTGCTGGTCGTGGGATCTACGCTGCGC
CGGATCCTGTTGAGGCTGTTCAGCGCTACCAGCAGGAGGGTTGGGAGGCATATGAGGCTAGGGTTGGCAAGCAGTGAGAATATTCTATGATCTGTCTTTT

>P_{H4}-2, 1067 bp insertion

GATGGAGAAAGAATAGAAGCTACTTACGGCCAGTCATGTTGATAGATGTGGATTAAGATTTTTAAAGTTGTAGAGACGTGGAGATGGATGAAGTTGAAAC
TCGTGGATAGAGACTTAGCTGGATGGAGAGAAGAGTGACTGGTAGCTGGGGCGAGAGATATTTCTATATCTAAGGGCAGAGCGCGGGGAGTTGCTGGAAT
CACGCACTAGCAGGGAGATCGACCAATCAGCGCGCGCAGACCTAGCGGCGGGTGTGATCTGCCGGTGCCTTGGCCGCGAACCTCTGATTCCGCTGGCAGA
GACTTGATCCTGCCGGGAGAGCCAATAACGACGGAGATCAAAGACGTTGAATTCTCAGCTGTGGCCTTTGAGACTATGTTCATCATTCTGGAGCCCTTGA
TCGAGCCACATTACAGCGTGGTGGTTGGGATCAAGAAGGGGTGATTGGCATTCTAGATCTGTGATCCTGGGCTCCCATTGGCTGGCGGGTGACATCTCGC
TTAGCGCTCCGGGAGCAAGGGTAACGTGCCTGCAGTTCTTTGAGTCTCCCAGCGAAACTGCCACGCCGAAATCACGGCTTATGGTGCACTGCTTTCCGAT
CGGTTTTCTGATCAAAAAGAATCCGCGCTAAGACCACCCAAACTCGCTGGCAGCACCCAGATCACGCGCCCGCCCTCGTCTCCTACAAATACCCTCCCCC
ACCCACCTCCTCCATCCTTCTTACAACTCACTCAGTCATCCACTCAACGCCCATCAAATCCATCGGTTTTATCCGACTTCCATCAATAACACACATCAAA
TCCATCACATAAACATCTAAACAATGGCTAGAACCAAGCAGACTAGTTCTAGAGCGGCCGCATCCACTCGTTCACTTGTTTGGTTCTTCTATTCTCACAC
TCGTTTTCCTCTAATGGGAATCAACATGCGTCGAGCTTTGGCCGTAGCTGCCATCGCAGCGTCCGCCAATGCAATGGTGAGCAAGGGCGAGGAGCTGTTC
ACCGGGGTGGTGCCCATCCTGGTCGAGCTGGACGGCGACGTAAACGGCCACAAGTTCAGCGTGTCCGGCGAGGGCGAGGGCGATGCCACCTACGGCAAGC
TGACCGAAGGTCTCAGTCGACTATGCCAGGAAGTACAAGGATTTCGTCATGGGCTTTGTTTCGACGAGGGCATTGTCGGAGATCAAATCGGACGTGACCA
CGGCTTCGGATGATGAGGACTTTGTGGTTTTCACCACCGGTGTCAATCTGTCGTCGAAGGGTGATAAGCTTGGACAGCAGTACCAGACACCTCAGTCAGC
GATCGGCCGCGGTGCGGACTTTATTATTGCTGGTCGTGGGATCTACGCTGCGCCGGATCCTGTTGAGGCTGTTCAGCGCTACCAGCAGGAGGGTTGGGAG
GCATATGAGGCTAGGGTTGGCAAGCAGTGAGAATATTCTATGATCTGTCTTTTTATATTTGTAGCAGAGTCGTGG

>P_{H4}-3, 1803 bp HR

TGCATTGAACGACCCCTCGAGGTCGACAGATGAAGGCGTTGATGGAGAAAGAATAGAAGCTACTTACGGCCAGTCATGTTGATAGATGTGGATTAAGATT
TTTAAAGTTGTAGAGACGTGGAGATGGATGAAGTTGAAACTCGTGGATAGAGACTTAGCTGGATGGAGAGAAGAGTGACTGGTAGCTGGGGCGAGAGATA
TTTCTATATCTAAGGGCAGAGCGCGGGGAGTTGCTGGAATCACGCACTAGCAGGGAGATCGACCAATCAGCGCGCGCAGACCTAGCGGCGGGTGTGATCT
GCCGGTGCCTTGGCCGCGAACCTCTGATTCCGCTGGCAGAGACTTGATCCTGCCGGGAGAGCCAATAACGACGGAGATCAAAGACGTTGAATTCTCAGCT
GTGGCCTTTGAGACTATGTTCATCATTCTGGAGCCCTTGATCGAGCCACATTACAGCGTGGTGGTTGGGATCAAGAAGGGGTGATTGGCATTCTAGATCT
GTGATCCTGGGCTCCCATTGGCTGGCGGGTGACATCTCGCTTAGCGCTCCGGGAGCAAGGGTAACGTGCCTGCAGTTCTTTGAGTCTCCCAGCGAAACTG
CCACGCCGAAATCACGGCTTATGGTGCACTGCTTTCCGATCGGTTTTCTGATCAAAAAGAATCCGCGCTAAGACCACCCAAACTCGCTGGCAGCACCCAG
ATCACCCGCCCGCCCTCGTCTCCTACAAATACCCTCCCCCACCCACCTCCTCCATCCTTCTTACAACTCACTCAGTCATCCACTCAACGCCCATCAAATC
CATCGGTTTTATCCGACTTCCATCAATAAAACACATCAAATTCCATCAAATAAACATCTAAACAATGGCTAGAACCAAGCAGACTAGTTCTAGAGCGGCC
GCATCCACTCGTTCACTTGTTTGGTTCTTCTATTCTCACACTCGTTTTCCTCTAATGGGAATCAACATGCGTCGAGCTTTGGCCGTAGCTGCCATCGCAG
CGTCCGCCAATGCAATGGTGAGCAAGGGCGAGGAGCTGTTCACCGGGGTGGTGCCCATCCTGGTCGAGCTGGACGGCGACGTAAACGGCCACAAGTTCAG
CGTGTCCGGCGAGGGCGAGGGCGATGCCACCTACGGCAAGCTGACCCTGAAGTTCATCTGCACCACCGGCAAGCTGCCCGTGCCCTGGCCCACCCTCGTG

ACCACCCTGACCTACGGCGTGCAGTGCTTCAGCCGCTACCCCGACCACATGAAGCAGCACGACTTCTTCAAGTCCGCCATGCCCGAAGGCTACGTCCAGG
AGCGCACCATCTTCTTCAAGGACGACGGCAACTACAAGACCCGCGCCGAGGTGAAGTTCGAGGGCGACACCCTGGTGAACCGCATCGAGCTGAAGGGCAT
CGACTTCAAGGAGGACGGCAACATCCTGGGGCACAAGCTGGAGTACAACTACAACAGCCACAACGTCTATATCATGGCCGACAAGCAGAAGAACGGCATC
AAGGTGAACTTCAAGATCCGCCACAACATCGAGGACGGCAGCGTGCAGCTCGCCGACCACTACCAGCAGAACACCCCCATCGGCGACGGCCCCGTGCTGC
TGCCCGACAACCACTACCTGAGCACCCAGTCCGCCCTGAGCAAAGACCCCAACGAGAAGCGCGATCACATGGTCCTGCTGGAGTTCGTGACCGCCGCCGG
GATCACTCTCGGCATGGACGAGCTGTACAAGTAAATCATTCCACTCAACATTCAGGCTCCTCTGCGCACGTAAAGTGCCAAAGGCAATACCCTGCGGCCG
CCACCGCGGTGGAGCTCGAATATTCTATGATCTGTCTTTTTATATTTGTAGCAGAGTCGTGGCTATATGACCCTTTTGTTACCCAGATACAGTATACAT

>P_{H4}-5, 148/569 bp insertion

ATTTACCCCTCTATCTGCATTGAACGACCATGTCTTCCAGATCGCAATTGAGATACTCTGAACGAGCACGAAACCATCCTAATGTGCTCGCGAAGAAGCT
GTTCGAAATAGCGGAGGCAAAGAAGACTAATGTCACTGTATCTGCCGATGTCACGACTACGAAGGAACTGCTGGATCTAGCTAACCGTAAGTTGGAACTT
ATTACTCTCAATTACACGCATCCGCGCATTCGAAGGTCAGGTTTTTTTTAAGCTAACAATCTATCTGCCCTATACAGGTCTCGGCCCCCATATCGCCGT
GATCAAAACCCACATTGACATCCTATCCGACTTCAGCGAAGAGACCATCTCCGGCCTGACGGCGCTCGCGAAGAAGCACAACTTCCTCATCTTTGAGGAC
CGCAAGTTCATCGATATCGGTAACACCGTCCAGAAGCAGTACCATGGTGGAGCTCTCCGGATATCTGAGTGGTCCCACATTATCAATTGCGCCATCCTCC
CCGGTGAGGGTATCGTCGAGGCTCTCGCCCAAACTGCTTCGTCGCCAGATTTCCCATACGGAAAGGAGAGGGGACTGCTGATTCTAGCAGAGATGACCTC
CAAGGGATCCCTTGCCACGGGAGAGTACAGCGTGTGATCTGCCGGTGCCTTGGCCGCGAACCTCTGATTCCGCTGGCAGAGACTTGATCCTGCCGGGAGA
GCCAATAACGACGGAGATCAAAGACGTTGAATTCTCAGCTGTGGCCTTTGAGACTATGTTCATCATTCTGGAGCCCTATGAAGCAGCACGACTTCTTCAA
GTCCGCCATGCCCGAAGGCTACGTCCAGGAGCGCACCATCTTCTTCAAGGACGACGGCAACTACAAGACCCGCGCCGAGGTGAAGTTCGAGGGCGACACC
CTGGTGAACCGCATCGAGCTGAAGGGCATCGACTTCAAGGAGGACGGCAACATCCTGGGGCACAAGCTGGAGTACAACTACAACAGCCACAACGTCTATA
TCATGGCCGACAAGCAGAAGAACGGCATCAAGGTGAACTTCAAGATCCGCCACAACATCGAGGACGGCAGCGTGCAGCTCGCCGACCACTACCAGCAGAA
CACCCCCATCGGCGACGGCCCCGTGCTGCTGCCCGACAACCACTACCTGAGCACCCAGTCCGCCCTGAGCAAAGACCCCAACGAGAAGCGCGATCACATG
GTCCTGCTGGAGTTCGTGACCGCCGCCGGGATCACTCTCGGCATGGACGAGCTGTACAAGTAAATCATTCCACTCAACATTCAGGCTCCTCTGCGCACGT
AAAGTGCCAAAGGCAATACCCTGCGGCCGCCACCGCGGTGGAGCTCGAATATTCTATGATCTGTCTTTTTATATTTGTAGCAGAGTCGTGGCTATATG

>P_{H4}-7, 1803 bp HR

TTACCCCTCTATCTGCATTGAACGACCCCTCGAGGTCGACAGATGAAGGCGTTGATGGAGAAAGAATAGAAGCTACTTACGGCCAGTCATGTTGATAGAT
GTGGATTAAGATTTTTAAAGTTGTAGAGACGTGGAGATGGATGAAGTTGAAACTCGTGGATAGAGACTTAGCTGGATGGAGAGAAGAGTGACTGGTAGCT
GGGGCGAGAGATATTTCTATATCTAAGGGCAGAGCGCGGGGAGTTGCTGGAATCACGCACTAGCAGGGAGATCGACCAATCAGCGCGCGCAGACCTAGCG
GCGGGTGTGATCTGCCGGTGCCTTGGCCGCGAACCTCTGATTCCGCTGGCAGAGACTTGATCCTGCCGGGAGAGCCAATAACGACGGAGATCAAAGACGT
TGAATTCTCAGCTGTGGCCTTTGAGACTATGTTCATCATTCTGGAGCCCTTGATCGAGCCACATTACAGCGTGGTGGTTGGGATCAAGAAGGGGTGATTG
GCATTCTAGATCTGTGATCCTGGGCTCCCATTGGCTGGCGGGTGACATCTCGCTTAGCGCTCCGGGAGCAAGGGTAACGTGCCTGCAGTTCTTTGAGTCT
CCCAGCGAAACTGCCACGCCGAAATCACGGCTTATGGTGCACTGCTTTCCGATCGGTTTTCTGATCAAAAAGAATCCGCGCTAAGACCACCCAAACTCGC
TGGCAGCAACCCAGATCACCCGCCCGCCCTCGTCTCCTACAAATACCCTCCCCCACCCACCTCCTCCATCCTTCTTACAACTCACTCAGTCATCCACTCA
ACGCCCATCAAATCCATCGGTTTTATCCGACTTCCATCAATAAAACACATCAAATCCATCAAATAAACATCTAAACAATGGCTAGAACCAAGCAGACTAG
TTCTAGAGCGGCCGCATCCACTCGTTCACTTGTTTGGTTCTTCTATTCTCACACTCGTTTTCCTCTAATGGGAATCAACATGCGTCGAGCTTTGGCCGTA
GCTGCCATCGCAGCGTCCGCCAATGCAATGGTGAGCAAGGGCGAGGAGCTGTTCACCGGGGTGGTGCCCATCCTGGTCGAGCTGGACGGCGACGTAAACG
GCCACAAGTTCAGCGTGTCCGGCGAGGGCGAGGGCGATGCCACCTACGGCAAGCTGACCCTGAAGTTCATCTGCACCACCGGCAAGCTGCCCGTGCCCTG
GCCCACCCTCGTGACCACCCTGACCTACGGCGTGCAGTGCTTCAGCCGCTACCCCGACCACATGAAGCAGCACGACTTCTTCAAGTCCGCCATGCCCGAA
GGCTACGTCCAGGAGCGCACCATCTTCTTCAAGGACGACGGCAACTACAAGACCCGCGCCGAGGTGAAGTTCGAGGGCGACACCCTGGTGAACCGCATCG
AGCTGAAGGGCATCGACTTCAAGGAGGACGGCAACATCCTGGGGCACAAGCTGGAGTACAACTACAACAGCCACAACGTCTATATCATGGCCGACAAGCA
GAAGAACGGCATCAAGGTGAACTTCAAGATCCGCCACAACATCGAGGACGGCAGCGTGCAGCTCGCCGACCACTACCAGCAGAACACCCCCATCGGCGAC
GGCCCCGTGCTGCTGCCCGACAACCACTACCTGAGCACCCAGTCCGCCCTGAGCAAAGACCCCAACGAGAAGCGCGATCACATGGTCCTGCTGGAGTTCG
TGACCGCCGCCGGGATCACTCTCGGCATGGACGAGCTGTACAAGTAAATCATTCCACTCAACATTCAGGCTCCTCTGCGCACGTAAAGTGCCAAAGGCAA
TACCCTGCGGCCGCCACCGCGGTGGAGCTCGAATATTCTATGATCTGTCTTTTTATATTTGTAGCAGAGTCGTGGCTATATGACCCTTTTGT

>P_{H4}-9, 128/165 bp insertion

TCTATCTGCATTGAACGACCATGTCTTCCAGATCGCAATTGAGATACTCTGAACGAGCACGAAACCATCCTAATGTGCTCGCGAAGAAGCTGTTCGAAAT
AGCGGAGGCAAAGAAGACTAATGTCACTGTATCTGCCGATGTCACGACTACGAAGGAACTGCTGGATCTAGCTAACCGTAAGTTGGAACTTATTACTCTC
AATTACACGCATCCGCGCATTCGAAGGTCAGGTTTTTTTTAAGCTAACAATCTATCTGCCCTATACAGGTCTCGGCCCCCATATCGCCGTGATCAAAAC
CCACATTGACATCCTATCCGACTTCAGCGAAGAGACCATCTCCGGCCTGACGGCGCTCGCGAAGAAGCACAACTTCCTCATCTTTGAGGACCGCAAGTTC
ATCGATATCGGTAACACCGTCCAGAAGCAGTACCATGGTGGAGCTCTCCGGATATCTGAGTGGTCCCACATTATCAATTGCGCCATCCTCCCCGGTGAGG
GTATCGTCGAGGCTCTCGCCCAAACTGCTTCGTCGCCAGATTTCCCATACGGAAAGGAGAGGGGACTGCTGATTCTAGCAGAGATGACCTCCAAGGGATC
CCTTGCCACGGGAGAGTACACCCTTCTTGATCCCAACCACCACGCTGTAATGTGGCTCGATCAAGGGCTCCAGAATGATGAACATAGTCTCAAAGGCCAC

AGCTGAGAATTCAACGTCTTTGATCTCCGTCGTTATTGGCTCTCCCGGGGGGCGAGAGATATTTCTATATCTAAGGGCAGAGCGCGGGGAGTTGCTGGAA
TCACGCACTAGCAGGGAGATCGACCAATCAGCGCGCGCAGACCTAGCGGCGGGTGTGATCTGCCGGTGCCTTGGCCGCGAACCTCTGATTCCGCTGGCAG
AGACTTGATCCTACGAAGGTCTCAGTCGACTATGCCAGGAAGTACAAGGATTTCGTCATGGGCTTTGTTTCGACGAGGGCATTGTCGGAGATCAAATCGG
ACGTGACCACGGCTTCGGATGATGAGGACTTTGTGGTTTTCACCACCGGTGTCAATCTGTCGTCGAAGGGTGATAAGCTTGGACAGCAGTACCAGACACC
TCAGTCAGCGATCGGCCGCGGTGCGGACTTTATTATTGCTGGTCGTGGGATCTACGCTGCGCCGGATCCTGTTGAGGCTGTTCAGCGCTACCAGCAGGAG
GGTTGGGAGGCATATGAGGCTAGGGTTGGCAAGCAGTGAGAATATTCTATGATCTGTCTTTTTATATTTGTAGCAGAGTCGTGGCTATATGACCCTTTTG
TTACCCAGATACAGTATACATACCACGTG

>P_{H4}-10, 338 bp insertion

ACCCCTCTATCTGCATTGAACGACCCCTCGAGGTCGACAGATGAAGGCGTTGATGGAGAAAGAATAGAAGCTACTTACGGCCAGTCATGTTGATAGATGT
GGATTAAGATTTTTAAAGTTGTAGAGACGTGGAGATGGATGAAGTTGAAACTCGTGGATAGAGACTTAGCTGGATGGAGAGAAGAGTGACTGGTAGCTGG
GGCGAGAGATATTTCTATATCTAAGGGCAGAGCGCGGGGAGTTGCTGGAATCACGCACTAGCAGGGAGATCGACCAATCAGCGCGCGCAGACCTAGCGGC
GGGTGTGATCTGCCGGTGCCTTGGCCGCGAACCTCTGATTCCGCTGGCAGAGACTTGATCCTACGAAGGTCTCAGTCGACTATGCCAGGAAGTACAAGGA
TTTCGTCATGGGCTTTGTTTCGACGAGGGCATTGTCGGAGATCAAATCGGACGTGACCACGGCTTCGGATGATGAGGACTTTGTGGTTTTCACCACCGGT
GTCAATCTGTCGTCGAAGGGTGATAAGCTTGGACAGCAGTACCAGACACCTCAGTCAGCGATCGGCCGCGGTGCGGACTTTATTATTGCTGGTCGTGGGA
TCTACGCTGCGCCGGATCCTGTTGAGGCTGTTCAGCGCTACCAGCAGGAGGGTTGGGAGGCATATGAGGCTAGGGTTGGCAAGCAGTGAGAATATTCTAT
GATCTGTCTTTTTATATTTGTAGCAGAGTCGTGGCTATATG

>P_{P2}-2, 38 bp insertion

AGATAGATAAGTACCATACTTACTTGCATGTATCGGCGGCCGGCACGGGCAGCTTGCCGGTGGTGCAGATGAACTTCAGCGAAGGTCTCAGTCGACTATG
CCAGGAAGTACAAGGATTTCGTCATGGGCTTTGTTTCGACGAGGGCATTGTCGGAGATCAAATCGGACGTGACCACGGCTTCGGATGATGAGGACTTTGT
GGTTTTCACCACCGGTGTCAATCTGTCGTCGAAGGGTGATAAGCTTGGACAGCAGTACCAGACACCTCAGTCAGCGATCGGCCGCGGTGCGGACTTTATT
ATTGCTGGTCGTGGGATCTACGCTGCGCCGGATCCTGTTGAGGCTGTTCAGCGCTACCAGCAGGAGGGTTGGGAGGCATATGAGGCTAGGGTTGGCAAGC
AGTGAGAATATTCTATGATCTGTCTTTTTATATTTGTAGCAGAGTCGTGGCTATATGACCCTTTTGTTACCCAGATACAGTATACATACCACGTG

>P_{P2}-4, 1857 bp HR

CTGCCCCTCCAAATTTACCCCTCTATCTGCATTGAACGACCCCTCGAGGTCGACGGAGCTTAAGAACTGCAAATCAGTCAGCAAAGATGCTACTTATCGG
GGTATCCTGTCATCAAAGGATGCATACACTCATCGTGAAGATTTTCCAGACCAATAGATACGAGTCTAAAAGGTGGGAAAGAAGGACATTCGTTCACCGC
CGATGGTTGGTACGACGAGGTAGCTTCATGACTTCAGCACGAGCGCGCCAGCCATTTATGGCAGATCAAAGCGTTGGGCAGCCGGTATTACTCGATGGCG
AGGTAACGCTCTCCGTAGAGGCTTCCACGGTCCAAGACAGACACTGGAGCGGAAAAGGGCGCGTCCAGAGTCTGGTTCCCGAGAATAATCAAGACCTCAC
GATAGCTCCAGCGAGGGCAGAAGGTGAGGATAGGAGTGAAGGCGGGGGAAGAAAGAGAATCACATCGACGACAACAGCCCAGTGAGGCTTCTAGAAGAGA
GCGTCACGTGAGCCGTGGCACGATTGGCGTTTCTGGTCTTTGTCGTCTGCCGCAGCGGATGACATAATACGGTATACGCACCAGCCACATCCACCTTCGC
TTACGGGCCCAGCACCAGCCACTGCTCCGCCGCACTTTCAGAAATGTGTGGGTTCGCGAATTAATTACCGGCGTCGTTAAATCTTCGTCCACCAAGCGAT
CTCGTCTTTCCGCATCTCAAACCCCCTCCGACGAACTTGACGTCTCAGTCTCCGCCCAACTTGCGGTTAAATCGATTGATTCTCCTGTTCGTATAACGTT
CATCTTCAGTAAGTCCAGCAGTCCTTTATTCTTCTCTCCAGTCTTCCGCAATCGTTTCCGGATGCGTTATTTCTGGATGAAGATCGATCGAATGCGACAG
TGCAGCTGACAAGTTCATCAATTTTTTTCAGAACCACATTCAAAATGAAGCACCTCGCCGCTTACTAGTTCTAGAGCGGCCGCATCCACTCGTTCACTTG
TTTGGGTCTTCTATTCTCACACTCGTTTTCCTCTAATGGGAATCAACATGCGTCGAGCTTTGGCCGTAGCTGCCATCGCAGCGTCCGCCAATGCAATGGT
GAGCAAGGGCGAGGAGCTGTTCACCGGGGTGGTGCCCATCCTGGTCGAGCTGGACGGCGACGTAAACGGCCACAAGTTCAGCGTGTCCGGCGAGGGCGAG
GGCGATGCCACCTACGGCAAGCTGACCCTGAAGTTCATCTGCACCACCGGCAAGCTGCCCGTGCCCTGGCCCACCCTCGTGACCACCCTGACCTACGGCG
TGCAGTGCTTCAGCCGCTACCCCGACCACATGAAGCAGCACGACTTCTTCAAGTCCGCCATGCCCGAAGGCTACGTCCAGGAGCGCACCATCTTCTTCAA
GGACGACGGCAACTACAAGACCCGCGCCGAGGTGAAGTTCGAGGGCGACACCCTGGTGAACCGCATCGAGCTGAAGGGCATCGACTTCAAGGAGGACGGC
AACATCCTGGGGCACAAGCTGGAGTACAACTACAACAGCCACAACGTCTATATCATGGCCGACAAGCAGAAGAACGGCATCAAGGTGAACTTCAAGATCC
GCCACAACATCGAGGACGGCAGCGTGCAGCTCGCCGACCACTACCAGCAGAACACCCCCATCGGCGACGGCCCCGTGCTGCTGCCCGACAACCACTACCT
GAGCACCCAGTCCGCCCTGAGCAAAGACCCCAACGAGAAGCGCGATCACATGGTCCTGCTGGAGTTCGTGACCGCCGCCGGGATCACTCTCGGCATGGAC
GAGCTGTACAAGTAAATCATTCCACTCAACATTCAGGCTCCTCTGCGCACGTAAAGTGCCAAAGGCAATACCCTGCGGCCGCCACCGCGGTGGAGCTCGA
ATATTCTATGATCTGTCTTTTTATATTTGTAGCAGAGTCGTGGCTATATGACCCTTTTGTTACCCAGATACAGTATACATACCA

>P_{P2}-5, 1857 bp HR

CCTGCCCCTCCAAATTTACCCCTCTATCTGCATTGAACGACCCCTCGAGGTCGACGGAGCTTAAGAACTGCAAATCAGTCAGCAAAGATGCTACTTATCG
GGGTATCCTGTCATCAAAGGATGCATACACTCATCGTGAAGATTTTCCAGACCAATAGATACGAGTCTAAAAGGTGGGAAAGAAGGACATTCGTTCACCG
CCGATGGTTGGTACGACGAGGTAGCTTCATGACTTCAGCACGAGCGCGCCAGCCATTTATGGCAGATCAAAGCGTTGGGCAGCCGGTATTACTCGATGGC
GAGGTAACGCTCTCCGTAGAGGCTTCCACGGTCCAAGACAGACACTGGAGCGGAAAAGGGCGCGTCCAGAGTCTGGTTCCCGAGAATAATCAAGACCTCA
CGATAGCTCCAGCGAGGGCAGAAGGTGAGGATAGGAGTGAAGGCGGGGGAAGAAAGAGAATCACATCGACGACAACAGCCCAGTGAGGCTTCTAGAAGAG

AGCGTCACGTGAGCCGTGGCACGATTGGCGTTTCTGGTCTTTGTCGTCTGCCGCAGCGGATGACATAATACGGTATACGCACCAGCCACATCCACCTTCG
CTTACGGGCCCAGCACCAGCCACTGCTCCGCCGCACTTTCAGAAATGTGTGGGTTCGCGAATTAATTACCGGCGTCGTTAAATCTTCGTCCACCAAGCGA
TCTCGTCTTTCCGCATCTCAAACCCCCTCCGACGAACTTGACGTCTCAGTCTCCGCCCAACTTGCGGTTAAATCGATTGATTCTCCTGTTCGTATAACGT
TCATCTTCAGTAAGTCCAGCAGTCCTTTATTCTTCTCTCCAGTCTTCCGCAATCGTTTCCGGATGCGTTATTTCTGGATGAAGATCGATCGAATGCGACA
GTGCAGCTGACAAGTTCATCAATTTTTTTCAGAACCACATTCAAAATGAAGCACCTCGCCGCTTACTAGTTCTAGAGCGGCCGCATCCACTCGTTCACTT
GTTTGGGTCTTCTATTCTCACACTCGTTTTCCTCTAATGGGAATCAACATGCGTCGAGCTTTGGCCGTAGCTGCCATCGCAGCGTCCGCCAATGCAATGG
TGAGCAAGGGCGAGGAGCTGTTCACCGGGGTGGTGCCCATCCTGGTCGAGCTGGACGGCGACGTAAACGGCCACAAGTTCAGCGTGTCCGGCGAGGGCGA
GGGCGATGCCACCTACGGCAAGCTGACCCTGAAGTTCATCTGCACCACCGGCAAGCTGCCCGTGCCCTGGCCCACCCTCGTGACCACCCTGACCTACGGC
GTGCAGTGCTTCAGCCGCTACCCCGACCACATGAAGCAGCACGACTTCTTCAAGTCCGCCATGCCCGAAGGCTACGTCCAGGAGCGCACCATCTTCTTCA
AGGACGACGGCAACTACAAGACCCGCGCCGAGGTGAAGTTCGAGGGCGACACCCTGGTGAACCGCATCGAGCTGAAGGGCATCGACTTCAAGGAGGACGG
CAACATCCTGGGGCACAAGCTGGAGTACAACTACAACAGCCACAACGTCTATATCATGGCCGACAAGCAGAAGAACGGCATCAAGGTGAACTTCAAGATC
CGCCACAACATCGAGGACGGCAGCGTGCAGCTCGCCGACCACTACCAGCAGAACACCCCCATCGGCGACGGCCCCGTGCTGCTGCCCGACAACCACTACC
TGAGCACCCAGTCCGCCCTGAGCAAAGACCCCAACGAGAAGCGCGATCACATGGTCCTGCTGGAGTTCGTGACCGCCGCCGGGATCACTCTCGGCATGGA
CGAGCTGTACAAGTAAATCATTCCACTCAACATTCAGGCTCCTCTGCGCACGTAAAGTGCCAAAGGCAATACCCTGCGGCCGCCACCGCGGTGGAGCTCG
AATATTCTATGATCTGTCTTTTTATATTTGTAGCAGAGTCGTGGCTATATGACCCTTTTGTTACCCAGATACAGTATACATACCACG

>P_{P2}-6, 1857 bp HR

GCATTGAACGACCCCTCGAGGTCGACGGAGCTTAAGAACTGCAAATCAGTCAGCAAAGATGCTACTTATCGGGGTATCCTGTCATCAAAGGATGCATACA
CTCATCGTGAAGATTTTCCAGACCAATAGATACGAGTCTAAAAGGTGGGAAAGAAGGACATTCGTTCACCGCCGATGGTTGGTACGACGAGGTAGCTTCA
TGACTTCAGCACGAGCGCGCCAGCCATTTATGGCAGATCAAAGCGTTGGGCAGCCGGTATTACTCGATGGCGAGGTAACGCTCTCCGTAGAGGCTTCCAC
GGTCCAAGACAGACACTGGAGCGGAAAAGGGCGCGTCCAGAGTCTGGTTCCCGAGAATAATCAAGACCTCACGATAGCTCCAGCGAGGGCAGAAGGTGAG
GATAGGAGTGAAGGCGGGGGAAGAAAGAGAATCACATCGACGACAACAGCCCAGTGAGGCTTCTAGAAGAGAGCGTCACGTGAGCCGTGGCACGATTGGC
GTTTCTGGTCTTTGTCGTCTGCCGCAGCGGATGACATAATACGGTATACGCACCAGCCACATCCACCTTCGCTTACGGGCCCAGCACCAGCCACTGCTCC
GCCGCACTTTCAGAAATGTGTGGGTTCGCGAATTAATTACCGGCGTCGTTAAATCTTCGTCCACCAAGCGATCTCGTCTTTCCGCATCTCAAACCCCCTC
CGACGAACTTGACGTCTCAGTCTCCGCCCAACTTGCGGTTAAATCGATTGATTCTCCTGTTCGTATAACGTTCATCTTCAGTAAGTCCAGCAGTCCTTTA
TTCTTCTCTCCAGTCTTCCGCAATCGTTTCCGGATGCGTTATTTCTGGATGAAGATCGATCGAATGCGACAGTGCAGCTGACAAGTTCATCAATTTTTTT
CAGAACCACATTCAAAATGAAGCACCTCGCCGCTTACTAGTTCTAGAGCGGCCGCATCCACTCGTTCACTTGTTTGGTTCTTCTATTCTCACACTCGTTT
TCCTCTAATGGGAATCAACATGCGTCGAGCTTTGGCCGTAGCTGCCATCGCAGCGTCCGCCAATGCAATGGTGAGCAAGGGCGAGGAGCTGTTCACCGGG
GTGGTGCCCATCCTGGTCGAGCTGGACGGCGACGTAAACGGCCACAAGTTCAGCGTGTCCGGCGAGGGCGAGGGCGATGCCACCTACGGCAAGCTGACCC
TGAAGTTCATCTGCACCACCGGCAAGCTGCCCGTGCCCTGGCCCACCCTCGTGACCACCCTGACCTACGGCGTGCAGTGCTTCAGCCGCTACCCCGACCA
CATGAAGCAGCACGACTTCTTCAAGTCCGCCATGCCCGAAGGCTACGTCCAGGAGCGCACCATCTTCTTCAAGGACGACGGCAACTACAAGACCCGCGCC
GAGGTGAAGTTCGAGGGCGACACCCTGGTGAACCGCATCGAGCTGAAGGGCATCGACTTCAAGGAGGACGGCAACATCCTGGGGCACAAGCTGGAGTACA
ACTACAACAGCCACAACGTCTATATCATGGCCGACAAGCAGAAGAACGGCATCAAGGTGAACTTCAAGATCCGCCACAACATCGAGGACGGCAGCGTGCA
GCTCGCCGACCACTACCAGCAGAACACCCCCATCGGCGACGGCCCCGTGCTGCTGCCCGACAACCACTACCTGAGCACCCAGTCCGCCCTGAGCAAAGAC
CCCAACGAGAAGCGCGATCACATGGTCCTGCTGGAGTTCGTGACCGCCGCCGGGATCACTCTCGGCATGGACGAGCTGTACAAGTAAATCATTCCACTCA
ACATTCAGGCTCCTCTGCGCACGTAAAGTGCCAAAGGCAATACCCTGCGGCCGCCACCGCGGTGGAGCTCGAATATTCTATGATCTGTCTTTTTATATTT
GTAGCAGAGTCGTGGCTATATGAC

>P_{P2}-7, 10 bp deletion, 17 bp insertion

TGCCCCTCCAAATTTACCCCTCTATCTGCATTGAACGACCATGTCTTCCAGATCGCAATTGAGATACTCTGAACGAGCACGAAACCATCCTAATGTGCTC
GCGAAGAAGCTGTTCGAAATAGCGGAGGCAAAGAAGACTAATGTCACTGTATCTGCCGATGTCACGACTACGAAGGAACTGCTGGATCTAGCTAACCGTA
AGTTGGAACTTATTACTCTCAATTACACGCATCCGCGCATTCGAAGGTCAGGTTTTTTTTTAAGCTAACAATCTATCTGCCCTATACAGGTCTCGGCCCC
CATATCGCCGTGATCAAAACCCACATTGACATCCTATCCGACTTCAGCGAAGAGACCATCTCCGGCCTGACGGCGCTCGCGAAGAAGCACAACTTCCTCA
TCTTTGAGGACCGCAAGTTCATCGATATCGGTAACACCGTCCAGAAGCAGTACCATGGTGGAGCTCTCCGGATATCTGAGTGGTCCCACATTATCAATTG
CGCCATCCTCCCCGGTGAGGGTATCGTCGAGGCTCTCGCCCAAACTGCTTCGTCGCCAGATTTCCCATACGGAAAGGAGAGGGGACTGCTGATTCTAGCA
GAGATGACCTCCAAGGGATCCCTTGCCACG______________AAAACCCTAAACCCTAACGAAGGTCTCAGTCGACTATGCCAGGAAGTACAAGGATT
TCGTCATGGGCTTTGTTTCGACGAGGGCATTGTCGGAGATCAAATCGGACGTGACCACGGCTTCGGATGATGAGGACTTTGTGGTTTTCACCACCGGTGT
CAATCTGTCGTCGAAGGGTGATAAGCTTGGACAGCAGTACCAGACACCTCAGTCAGCGATCGGCCGCGGTGCGGACTTTATTATTGCTGGTCGTGGGATC
TACGCTGCGCCGGATCCTGTTGAGGCTGTTCAGCGCTACCAGCAGGAGGGTTGGGAGGCATATGAGGCTAGGGTTGGCAAGCAGTGAGAATATTCTATGA
TCTGTCTTTTTATATTTGTAGCAGAGTCGTGGCTATATGACCCTTTTGTTACCCAGATACAGTATACATACCACG

>P_{P2}-9, 58 bp insertion

GGAGAAAGGATAGGACAGAATATTTTCCATCCTGCCCCTCCAAATTTACCCCTCTATCTGCATTGAACGACCATGTCTTCCAGATCGCAATTGAGATACT

CTGAACGAGCACGAAACCATCCTAATGTGCTCGCGAAGAAGCTGTTCGAAATAGCGGAGGCAAAGAAGACTAATGTCACTGTATCTGCCGATGTCACGAC
TACGAAGGAACTGCTGGATCTAGCTAACCGTAAGTTGGAACTTATTACTCTCAATTACACGCATCCGCGCATTCGAAGGTCAGGTTTTTTTTTAAGCTAA
CAATCTATCTGCCCTATACAGGTCTCGGCCCCCATATCGCCGTGATCAAAACCCACATTGACATCCTATCCGACTTCAGCGAAGAGACCATCTCCGGCCT
GACGGCGCTCGCGAAGAAGCACAACTTCCTCATCTTTGAGGACCGCAAGTTCATCGATATCGGTAACACCGTCCAGAAGCAGTACCATGGTGGAGCTCTC
CGGATATCTGAGTGGTCCCACATTATCAATTGCGCCATCCTCCCCGGTGAGGGTATCGTCGAGGCTCTCGCCCAAACTGCTTCGTCGCCAGATTTCCCAT
ACGGAAAGGAGAGGGGACTGCTGATTCTAGCAGAGATGACCTCCAAGGGATCCCTTGCCACGGGAGAGTACATGCAGATGAACTTCAGGGTCAGCTTGCC
GTAGGTGGCATCGCCCTCGCCCTCGCCGGACGAAGGTCTCAGTCGACTATGCCAGGAAGTACAAGGATTTCGTCATGGGCTTTGTTTCGACGAGGGCATT
GTCGGAGATCAAATCGGACGTGACCACGGCTTCGGATGATGAGGACTTTGTGGTTTTCACCACCGGTGTCAATCTGTCGTCGAAGGGTGATAAGCTTGGA
CAGCAGTACCAGACACCTCAGTCAGCGATCGGCCGCGGTGCGGACTTTATTATTGCTGGTCGTGGGATCTACGCTGCGCCGGATCCTGTTGAGGCTGTTC
AGCGCTACCAGCAGGAGGGTTGGGAGGCATATGAGGCTAGGGTTGGCAAGCAGTGAGAATATTCTATGATC

>P_{S8}-1, 121, 139 bp insertion

CCTGCCCCTCCAAATTTACCCCTCTATCTGCATTGAACGACCATGTCTTCCAGATCGCAATTGAGATACTCTGAACGAGCACGAAACCATCCTAATGTGC
TCGCGAAGAAGCTGTTCGAAATAGCGGAGGCAAAGAAGACTAATGTCACTGTATCTGCCGATGTCACGACTACGAAGGAACTGCTGGATCTAGCTAACCG
TAAGTTGGAACTTATTACTCTCAATTACACGCATCCGCGCATTCGAAGGTCAGGTTTTTTTTTAAGCTAACAATCTATCTGCCCTATACAGGTCTCGGCC
CCCATATCGCCGTGATCAAAACCCACATTGACATCCTATCCGACTTCAGCGAAGAGACCATCTCCGGCCTGACGGCGCTCGCGAAGAAGCACAACTTCCT
CATCTTTGAGGACCGCAAGTTCATCGATATCGGTAACACCGTCCAGAAGCAGTACCATGGTGGAGCTCTCCGGATATCTGAGTGGTCCCACATTATCAAT
TGCGCCATCCTCCCCGGTGAGGGTATCGTCGAGGCTCTCGCCCAAACTGCTTCGTCGCCAGATTTCCCATACGGAAAGGAGAGGGGACTGCTGATTCTAG
CAGAGATGACCTCCAAGGGATCCCTTGCCACGGGAGAGTACGGAGCTAGATAGATAAGTACCATACTTACTTGCATGTATCGGCGGCCAGCCAATCGAAG
TGGGGCACCGTCATCGCGGGCCGCATTCGTGAATTCAGCGAATTTTCGATGCATTACAACTACAAGGGAAGCTGAGCTCACGTATGCAAAGTGACGGGCA
GGGATTTGCTGTCCTTGAGTTCTCCGAGATATCGGGAAAGTTGGGGAAGGGTGATGCGAATATAGTCTTGTTGGCTAAGTTGAAGGTGGACTCGTGGAGA
G________CTCAGTCGACTATGCCAGGAAGTACAAGGATTTCGTCATGGGCTTTGTTTCGACGAGGGCATTGTCGGAGATCAAATCGGACGTGACCACG
GCTTCGGATGATGAGGACTTTGTGGTTTTCACCACCGGTGTCAATCTGTCGTCGAAGGGTGATAAGCTTGGACAGCAGTACCAGACACCTCAGTCAGCGA
TCGGCCGCGGTGCGGACTTTATTATTGCTGGTCGTGGGATCTACGCTGCGCCGGATCCTGTTGAGGCTGTTCAGCGCTACCAGCAGGAGGGTTGGGAGGC
ATATGAGGCTAGGGTTGGCAAGCAGTGAGAATATTCTATGATCTGTCTTTTTATATTTGTAGCAGAGTCGTGGCTATATGACCCTTTTGTTACCCAGATA
CAGTATACATACCACG

>P_{S8}-2, 1816 bp HR

CCCTCCAAATTTACCCCTCTATCTGCATTGAACGACCCCTCGAGGTCGACGCGATCGTGAGAAAGGAGAAGGCCAAGAAGCAGCAGCAGAAGCCCAATCT
GTCGGGCAAACCATAGAGAGAGAGCTCTCGTGTCTGGTGGTTGAGGCATGTTTGATTGTAGATGGAGTGTTGAGTTTCATACCCAGTTAGACGATGTTCT
GATAGTAAATAGTGGCAGTTCTTTCTGCATGAATGTGATAAATAATCAGTGAATAATGATAATAAGGCCGTTGTGTTTCGAGGTTTAACTCTCAGTATAG
TAGTAGTACTGTAAGTACTTAGAAAAATCCCAGTACGAATTGGGAATGCTCGGCTGGCGGGCGTGTTGTGCGCTAGTCCATATTAGGGTTAGTGCGCACT
TTCGGGTAGGGCATTTTCGACAAGCGAGCCACACTGCGAAATTCAAGACAGCCGACGACAAGCACTTAACAATCGACCACCCACCCACATTGCCCGCGCC
GTTGTTCTGGTGAAGGACTGCAGGTTACGAGGTTAGTATATTTGATGCCGTTGTCGCCGTTTGCATTCTCTCGAGACGAAATTGGACAACTTCGTGGTCG
GGTGATACTCCGCGGCGGATTGATGGTGTCGATATTTCGTGAAGGAACCAAAGGTCAACAGAGCTTGGGTCAAAAGAGTCCTCGCGAGGAAAGCAGAAGA
AAGAGAAAAGAGAGACCTCGCGATCTATCACCTGTCTACGAAGTTTCCTACGTTCATCATCGAGAGGATTTTTTCTGCCGTCGTTGTGCCTCAAGAGAAT
CGCTCACTGACATCTGAATTTCCAACTCTACAGTAGTCCAGTCCATCAACCGCAAATATGGGTATTTCTCGCGACTCCCGTCACAAGCGCTCGGCCACCG
GTGCGAAGAGGGCCTTCTACTAGTTCTAGAGCGGCCGCATCCACTCGTTCACTTGTTTGGTTCTTCTATTCTCACACTCGTTTTCCTCTAATGGGAATCA
ACATGCGTCGAGCTTTGGCCGTAGCTGCCATCGCAGCGTCCGCCAATGCAATGGTGAGCAAGGGCGAGGAGCTGTTCACCGGGGTGGTGCCCATCCTGGT
CGAGCTGGACGGCGACGTAAACGGCCACAAGTTCAGCGTGTCCGGCGAGGGCGAGGGCGATGCCACCTACGGCAAGCTGACCCTGAAGTTCATCTGCACC
ACCGGCAAGCTGCCCGTGCCCTGGCCCACCCTCGTGACCACCCTGACCTACGGCGTGCAGTGCTTCAGCCGCTACCCCGACCACATGAAGCAGCACGACT
TCTTCAAGTCCGCCATGCCCGAAGGCTACGTCCAGGAGCGCACCATCTTCTTCAAGGACGACGGCAACTACAAGACCCGCGCCGAGGTGAAGTTCGAGGG
CGACACCCTGGTGAACCGCATCGAGCTGAAGGGCATCGACTTCAAGGAGGACGGCAACATCCTGGGGCACAAGCTGGAGTACAACTACAACAGCCACAAC
GTCTATATCATGGCCGACAAGCAGAAGAACGGCATCAAGGTGAACTTCAAGATCCGCCACAACATCGAGGACGGCAGCGTGCAGCTCGCCGACCACTACC
AGCAGAACACCCCCATCGGCGACGGCCCCGTGCTGCTGCCCGACAACCACTACCTGAGCACCCAGTCCGCCCTGAGCAAAGACCCCAACGAGAAGCGCGA
TCACATGGTCCTGTTGGAGTTCGTGACCGCCGCCGGGATCACTCTCGGCATGGACGAGCTGTACAAGTAAATCATTCCACTCAACATTCAGGCTCCTCTG
CGCACGTAAAGTGCCAAAGGCAATACCCTGCGGCCGCCACCGCGGTGGAGCTCGAATATTCTATGATCTGTCTTTTTATATTTGTAGCAGAGTCGTGGCT
ATATGACCCTTTTGTTACCCAGATACAGTATACATACCACGTG

>P_{S8}-3, 149 bp insertion

CTGCCCCTCCAAATTTACCCCTCTATCTGCATTGAACGACCCCTCGAGGTCGACGCGATCGTGAGAAAGGAGAAGGCATCCACTCGTTCACTTGTTTGGT
TCTTCTATTCTCACACTCGTTTTCCTCTAATGGGAATCAACATGCGTCGAGCTTTGGCCGTAGCTGCCATCGCAGCGTCCGCCAATGCAACGAAGGTCTC
AGTCGACTATGCCAGGAAGTACAAGGATTTCGTCATGGGCTTTGTTTCGACGAGGGCATTGTCGGAGATCAAATCGGACGTGACCACGGCTTCGGATGAT

GAGGACTTTGTGGTTTTCACCACCGGTGTCAATCTGTCGTCGAAGGGTGATAAGCTTGGACAGCAGTACCAGACACCTCAGTCAGCGATCGGCCGCGGTG
CGGACTTTATTATTGCTGGTCGTGGGATCTACGCTGCGCCGGATCCTGTTGAGGCTGTTCAGCGCTACCAGCAGGAGGGTTGGGAGGCATATGAGGCTAG
GGTTGGCAAGCAGTGAGAATATTCTATGATCTGTCTTTTTATATTTGTAGCAGAGTCGTGGCTATATGACCCTTTTGTTACCCAGATACAGTATACATAC
CACGTGATGATTTCAACTAGGTATTTTCCAAACAGGAAATAAAGAAGCTCACTTTCTAACCCATTTCAAATCCCAATATGCCGAATAGCAAAAAGAAAAA
GACCTCACTTCCTCCGACCTCTTCTGTACCATTCAAGGGAAGCTGAGCTCACGTATGCAAAGTGACGGGCAGGGATTTGCTGTCCTTGAGTTCTCCGAGA
TATCGGGAAAGTTGGGGAAGGGTGATGCGAATATAGTCTTGTTGGCTAAGTT

>P_{S8}-4, 246 bp insertion

CCCTCCAAATTTACCCCTCTATCTGCATTGAACGACCATGTCTTCCAGATCGCAATTGAGATACTCTGAACGAGCACGAAACCATCCTAATGTGCTCGCG
AAGAAGCTGTTCGAAATAGCGGAGGCAAAGAAGACTAATGTCACTGTATCTGCCGATGTCACGACTACGAAGGAACTGCTGGATCTAGCTAACCGTAAGT
TGGAACTTATTACTCTCAATTACACGCATCCGCGCATTCGAAGGTCAGGTTTTTTTTTAAGCTAACAATCTATCTGCCCTATACAGGTCTCGGCCCCCAT
ATCGCCGTGATCAAAACCCACATTGACATCCTATCCGACTTCAGCGAAGAGACCATCTCCGGCCTGACGGCGCTCGCGAAGAAGCACAACTTCCTCATCT
TTGAGGACCGCAAGTTCATCGATATCGGTAACACCGTCCAGAAGCAGTACCATGGTGGAGCTCTCCGGATATCTGAGTGGTCCCACATTATCAATTGCGC
CATCCTCCCCGGTGAGGGTATCGTCGAGGCTCTCGCCCAAACTGCTTCGTCGCCAGATTTCCCATACGGAAAGGAGAGGGGACTGCTGATTCTAGCAGAG
ATGACCTCCAAGGGATCCCTTGCCACGGGAGAGTACAGGCTCCCCGATCGTCGATCCGCTGGCCAATGTATACGACTTGAAAACACTACGACTCACTATA
GGTGCCACGGGAGAGTACACGAGTTTTAGAGCTAGAAATAGCAAGTTAAAATAAGGCTAGTCCGTTATCAACTTGAAAAAGTGGCACCGAGTCGGTGCTC
TCTGGTCTGTCGGGGGCGACAAGTGAGCTTTTCATCGTCGCTATCGTCCGCGGACCGCTTGCTTGACGTGCTATGACGGGAAGCGAAGGTCTCAGTCGAC
TATGCCAGGAAGTACAAGGATTTCGTCATGGGCTTTGTTTCGACGAGGGCATTGTCGGAGATCAAATCGGACGTGACCACGGCTTCGGATGATGAGGACT
TTGTGGTTTTCACCACCGGTGTCAATCTGTCGTCGAAGGGTGATAAGCTTGGACAGCAGTACCAGACACCTCAGTCAGCGATCGGCCGCGGTGCGGACTT
TATTATTGCTGGTCGTGGGATCTACGCTGCGCCGGATCCTGTTGAGGCTGTTCAGCGCTACCAGCAGGAGGGTTGGGAGGCATATGAGGCTAGGGTTGGC
AAGCAGTGAGAATATTCTATGATCTGTCTTTTTATATTTGTAGCAGAGTCGTGGCTATATGACCCTTTTGTTACCCAGATACAGTATA

>P_{S8}-5, 290 bp insertion

CCTCCAAATTTACCCCTCTATCTGCATTGAACGACCATGTCTTCCAGATCGCAATTGAGATACTCTGAACGAGCACGAAACCATCCTAATGTGCTCGCGA
AGAAGCTGTTCGAAATAGCGGAGGCAAAGAAGACTAATGTCACTGTATCTGCCGATGTCACGACTACGAAGGAACTGCTGGATCTAGCTAACCGTAAGTT
GGAACTTATTACTCTCAATTACACGCATCCGCGCATTCGAAGGTCAGGTTTTTTTTTAAGCTAACAATCTATCTGCCCTATACAGGTCTCGGCCCCCATA
TCGCCGTGATCAAAACCCACATTGACATCCTATCCGACTTCAGCGAAGAGACCATCTCCGGCCTGACGGCGCTCGCGAAGAAGCACAACTTCCTCATCTT
TGAGGACCGCAAGTTCATCGATATCGGTAACACCGTCCAGAAGCAGTACCATGGTGGAGCTCTCCGGATATCTGAGTGGTCCCACATTATCAATTGCGCC
ATCCTCCCCGGTGAGGGTATCGTCGAGGCTCTCGCCCAAACTGCTTCGTCGCCAGATTTCCCATACGGAAAGGAGAGGGGACTGCTGATTCTAGCAGAGA
TGACCTCCAAGGGATCCCTTGCCACGGGAGAGTACACGTCTGAATCCGGATACCGCTCGCCAGCTGATCTTTAATCTGTCGCGTTATCTGCGCTATAACA
TTGAATTAAAAGACGATGAGCAAATCGATATCAAAAAAGAGCTGTATCAAATTAAAGACTATATTGCCATTGAGCAGGCCCGCTTTGGCGACAAGCTCAC
GGTTATCTATGATATTGATGAAGAGGTGAATTGCTGCATTCCCAGCCTGCTGATCCAGCCGTTGGTGGAGAATGCCATTGTCCACGGTATCCAGCCTTGC
AAAGGTAAAGGCGTTGTCACCATCAGCGCGAAGGTCTCAGTCGACTATGCCAGGAAGTACAAGGATTTCGTCATGGGCTTTGTTTCGACGAGGGCATTGT
CGGAGATCAAATCGGACGTGACCACGGCTTCGGATGATGAGGACTTTGTGGTTTTCACCACCGGTGTCAATCTGTCGTCGAAGGGTGATAAGCTTGGACA
GCAGTACCAGACACCTCAGTCAGCGATCGGCCGCGGTGCGGACTTTATTATTGCTGGTCGTGGGATCTACGCTGCGCCGGATCCTGTTGAGGCTGTTCAG
CGCTACCAGCAGGAGGGTTGGGAGGCATATGAGGCTAGGGTTGGCAAGCAGTGAGAATATTCTATGATCTGTCTTTTTATATTTGTAGCAGAGTCGTGGC
TATATGACCCTTTTGTTACCCAGATACAGTATACATA

>P_{S8}-9, 1816 bp HR

TTTACCCCTCTATCTGCATTGAACGACCCCTCGAGGTCGACGCGATCGTGAGAAAGGAGAAGGCCAAGAAGCAGCAGCAGAAGCCCAATCTGTCGGGCAA
ACCATAGAGAGAGAGCTCTCGTGTCTGGTGGTTGAGGCATGTTTGATTGTAGATGGAGTGTTGAGTTTCATACCCAGTTAGACGATGTTCTGATAGTAAA
TAGTGGCAGTTCTTTCTGCATGAATGTGATAAATAATCAGTGAATAATGATAATAAGGCCGTTGTGTTTCGAGGTTTAACTCTCAGTATAGTAGTAGTAC
TGTAAGTACTTAGAAAAATCCCAGTACGAATTGGGAATGCTCGGCTGGCGGGCGTGTTGTGCGCTAGTCCATATTAGGGTTAGTGCGCACTTTCGGGTAG
GGCATTTTCGACAAGCGAGCCACACTGCGAAATTCAAGACAGCCGACGACAAGCACTTAACAATCGACCACCCACCCACATTGCCCGCGCCGTTGTTCTG
GTGAAGGACTGCAGGTTACGAGGTTAGTATATTTGATGCCGTTGTCGCCGTTTGCATTCTCTCGAGACGAAATTGGACAACTTCGTGGTCGGGTGATACT
CCGCGGCGGATTGATGGTGTCGATATTTCGTGAAGGAACCAAAGGTCAACAGAGCTTGGGTCAAAAGAGTCCTCGCGAGGAAAGCAGAAGAAAGAGAAAA
GAGAGACCTCGCGATCTATCACCTGTCTACGAAGTTTCCTACGTTCATCATCGAGAGGATTTTTTCTGCCGTCGTTGTGCCTCAAGAGAATCGCTCACTG
ACATCTGAATTTCCAACTCTACAGTAGTCCAGTCCATCAACCGCAAATATGGGTATTTCTCGCGACTCCCGTCACAAGCGCTCGGCCACCGGTGCGAAGA
GGGCCTTCTACTAGTTCTAGAGCGGCCGCATCCACTCGTTCACTTGTTTGGTTCTTCTATTCTCACACTCGTTTTCCTCTAATGGGAATCAACATGCGTC
GAGCTTTGGCCGTAGCTGCCATCGCAGCGTCCGCCAATGCAATGGTGAGCAAGGGCGAGGAGCTGTTCACCGGGGTGGTGCCCATCCTGGTCGAGCTGGA
CGGCGACGTAAACGGCCACAAGTTCAGCGTGTCCGGCGAGGGCGAGGGCGATGCCACCTACGGCAAGCTGACCCTGAAGTTCATCTGCACCACCGGCAAG
CTGCCCGTGCCCTGGCCCACCCTCGTGACCACCCTGACCTACGGCGTGCAGTGCTTCAGCCGCTACCCCGACCACATGAAGCAGCACGACTTCTTCAAGT
CCGCCATGCCCGAAGGCTACGTCCAGGAGCGCACCATCTTCTTCAAGGACGACGGCAACTACAAGACCCGCGCCGAGGTGAAGTTCGAGGGCGACACCCT

GGTGAACCGCATCGAGCTGAAGGGCATCGACTTCAAGGAGGACGGCAACATCCTGGGGCACAAGCTGGAGTACAACTACAACAGCCACAACGTCTATATC
ATGGCCGACAAGCAGAAGAACGGCATCAAGGTGAACTTCAAGATCCGCCACAACATCGAGGACGGCAGCGTGCAGCTCGCCGACCACTACCAGCAGAACA
CCCCCATCGGCGACGGCCCCGTGCTGCTGCCCGACAACCACTACCTGAGCACCCAGTCCGCCCTGAGCAAAGACCCCAACGAGAAGCGCGATCACATGGT
CCTGCTGGAGTTCGTGACCGCCGCCGGGATCACTCTCGGCATGGACGAGCTGTACAAGTAAATCATTCCACTCAACATTCAGGCTCCTCTGCGCACGTAA
AGTGCCAAAGGCAATACCCTGCGGCCGCCACCGCGGTGGAGCTCGAATATTCTATGATCTGTCTTTTTATATTTGTAGCAGAGTCGTGGCTATATGACCC
TTTTGTTACCCAGATACAGTATACATACCACGTG

>P_{S8}-10, 1816 bp HR

CCCCTCCAAATTTACCCCTCTATCTGCATTGAACGACCCCTCGAGGTCGACGCGATCGTGAGAAAGGAGAAGGCCAAGAAGCAGCAGCAGAAGCCCAATC
TGTCGGGCAAACCATAGAGAGAGAGCTCTCGTGTCTGGTGGTTGAGGCATGTTTGATTGTAGATGGAGTGTTGAGTTTCATACCCAGTTAGACGATGTTC
TGATAGTAAATAGTGGCAGTTCTTTCTGCATGAATGTGATAAATAATCAGTGAATAATGATAATAAGGCCGTTGTGTTTCGAGGTTTAACTCTCAGTATA
GTAGTAGTACTGTAAGTACTTAGAAAAATCCCAGTACGAATTGGGAATGCTCGGCTGGCGGGCGTGTTGTGCGCTAGTCCATATTAGGGTTAGTGCGCAC
TTTCGGGTAGGGCATTTTCGACAAGCGAGCCACACTGCGAAATTCAAGACAGCCGACGACAAGCACTTAACAATCGACCACCCACCCACATTGCCCGCGC
CGTTGTTCTGGTGAAGGACTGCAGGTTACGAGGTTAGTATATTTGATGCCGTTGTCGCCGTTTGCATTCTCTCGAGACGAAATTGGACAACTTCGTGGTC
GGGTGATACTCCGCGGCGGATTGATGGTGTCGATATTTCGTGAAGGAACCAAAGGTCAACAGAGCTTGGGTCAAAAGAGTCCTCGCGAGGAAAGCAGAAG
AAAGAGAAAAGAGAGACCTCGCGATCTATCACCTGTCTACGAAGTTTCCTACGTTCATCATCGAGAGGATTTTTTCTGCCGTCGTTGTGCCTCAAGAGAA
TCGCTCACTGACATCTGAATTTCCAACTCTACAGTAGTCCAGTCCATCAACCGCAAATATGGGTATTTCTCGCGACTCCCGTCACAAGCGCTCGGCCACC
GGTGCGAAGAGGGCCTTCTACTAGTTCTAGAGCGGCCGCATCCACTCGTTCACTTGTTTGGTTCTTCTATTCTCACACTCGTTTTCCTCTAATGGGAATC
AACATGCGTCGAGCTTTGGCCGTAGCTGCCATCGCAGCGTCCGCCAATGCAATGGTGAGCAAGGGCGAGGAGCTGTTCACCGGGGTGGTGCCCATCCTGG
TCGAGCTGGACGGCGACGTAAACGGCCACAAGTTCAGCGTGTCCGGCGAGGGCGAGGGCGATGCCACCTACGGCAAGCTGACCCTGAAGTTCATCTGCAC
CACCGGCAAGCTGCCCGTGCCCTGGCCCACCCTCGTGACCACCCTGACCTACGGCGTGCAGTGCTTCAGCCGCTACCCCGACCACATGAAGCAGCACGAC
TTCTTCAAGTCCGCCATGCCCGAAGGCTACGTCCAGGAGCGCACCATCTTCTTCAAGGACGACGGCAACTACAAGACCCGCGCCGAGGTGAAGTTCGAGG
GCGACACCCTGGTGAACCGCATCGAGCTGAAGGGCATCGACTTCAAGGAGGACGGCAACATCCTGGGGCACAAGCTGGAGTACAACTACAACAGCCACAA
CGTCTATATCATGGCCGACAAGCAGAAGAACGGCATCAAGGTGAACTTCAAGATCCGCCACAACATCGAGGACGGCAGCGTGCAGCTCGCCGACCACTAC
CAGCAGAACACCCCCATCGGCGACGGCCCCGTGCTGCTGCCCGACAACCACTACCTGAGCACCCAGTCCGCCCTGAGCAAAGACCCCAACGAGAAGCGCG
ATCACATGGTCCTGCTGGAGTTCGTGACCGCCGCCGGGATCACTCTCGGCATGGACGAGCTGTACAAGTAAATCATTCCACTCAACATTCAGGCTCCTCT
GCGCACGTAAAGTGCCAAAGGCAATACCCTGCGGCCGCCACCGCGGTGGAGCTCGAATATTCTATGATCTGTCTTTTTATATTTGTAGCAGAGTCGTGGC
TATATGACCCTTTTGTTACCCAGATACAGTATACATACCACG

>P_{S8}-11, >545 bp insertion

TACCCCTCTATCTGCATTGAACGACCCCTCGAGGTCGACGCGATCGTGAGAAAGGAGAAGGCCAAGAAGCAGCAGCAGAAGCCCAATCTGTCGGGCAAAC
CATAGAGAGAGAGCTCTCGTGTCTGGTGGTTGAGGCATGTTTGATTGTAGATGGAGTGTTGAGTTTCATACCCAGTTAGACGATGTTCTGATAGTAAATA
GTGGCAGTTCTTTCTGCATGAATGTGATAAATAATCAGTGAATAATGATAATAAGGCCGTTGTGTTTCGAGGTTTAACTCTCAGTATAGTAGTAGTACTG
TAAGTACTTAGAAAAATCCCAGTACGAATTGGGAATGCTCGGCTGGCGGGCGTGTTGTGCGCTAGTCCATATTAGGGTTAGTGCGCACTTTCGGGTAGGG
CATTTTCGACAAGCGAGCCACACTGCGAAATTCAAGACAGCCGACGACAAGCACTTAACAATCGACCACCCACCCACATTGCCCGCGCCGTTGTTCTGGT
GAAGGACTGCAGGTTACGAGGTTAGTATATTTGATGCCGTTGTCGCCGTTTGCATTCTCTCGAGACGAAATCGAAGGTCTCAGTCGACTATGCCAGGAAG
TACAAGGATTTCGTCATGGGCTTTGTTTCGACGAGGGCATTGTCGGAGATCAAATCGGACGTGACCACGGCTTCGGATGATGAGGACTTTGTGGTTTTCA
CCACCGGTGTCAATCTGTCGTCGAAGGGTGATAAGCTTGGACAGCAGTACCAGACACCTCAGTCAGCGATCGGCCGCGGTGCGGACTTTATTATTGCTGG
TCGTGGGATCTACGCTGCGCCGGATCCTGTTGAGGCTGTTCAGCGCTACCAGCAGGAGGGTTGGGAGGCATATGAGGCTAGGGTTGGCAAGCAGTGAGAA
TATTCTATGATCTGCCTTTTTATATTTGTAGCAGAATCGTGGCTATATGACCCTTTTGTTACCCAGATACCGTATACTTACCACGTGATGATTTCAACTA
GGTATTTTCCAA

>P_{tub}-1, 1583 bp HR

ACCCCTCTATCTGCATTGAACGACCCCTCGAGGTCGACAGTACTGGGGCATCGACGACTTGTCACGGGAGATATTTCTGTATTTTTAGGACAAAGCTGAC
GGGCATTTTATATCGAGGATCTCGGGTGACCATCCTCTCGGGGTAATTATGTTTGGGGTGGGTTAGTTAGAGCGGCTAAGTACCTACTGAACACAGCGCC
CACGAAGACCACTGGTTGATGACAAATCATAACAAATAAATACTTGACCAATTGTCGAGTATGTGCCATCAATGGGATGTCCTCAAAATGAGTATATTTC
TGGCCATGAACGTAGTTATTAATTTCTCTGTCTCCAATGTAACCAAGAGTTCCTCTATCCAGGTCGTTCCTGCATGGACAACGCGGGCTGGTCCCGTGAC
GTGGCACGAACCGGCGCGATACCGGATTGAGCGCGCCCCCTGCCCGGCCTCTTGTTGTCCTGTTGACTCCTGAACTAACCTTCTTAGCCGCACAACAAGG
CGTCTCCTCACCGCCGACTTCTTCCCAACTTCATCCTCCATCGCTGTCCTTTCTCCCAGAACTCCTCTTGTCCTGGGTTTCAATCCTCTCCTCATCCTCC
CGTCCATTCCAACTCTCTTATTTACCGGACCTGTCACCTTCCTGCCACCATGCGTGAAATCGTACGTTTGTTGACTAGTTCTAGAGCGGCCGCATCCACT
CGTTCACTTGTTTGGTTCTTCTATTCTCACACTCGTTTTCCTCTAATGGGAATCAACATGCGTCGAGCTTTGGCCGTAGCTGCCATCGCAGCGTCCGCCA
ATGCAATGGTGAGCAAGGGCGAGGAGCTGTTCACCGGGGTGGTGCCCATCCTGGTCGAGCTGGACGGCGACGTAAACGGCCACAAGTTCAGCGTGTCCGG
CGAGGGCGAGGGCGATGCCACCTACGGCAAGCTGACCCTGAAGTTCATCTGCACCACCGGCAAGCTGCCCGTGCCCTGGCCCACCCTCGTGACCACCCTG

ACCTACGGCGTGCAGTGCTTCAGCCGCTACCCCGACCACATGAAGCAGCACGACTTCTTCAAGTCCGCCATGCCCGAAGGCTACGTCCAGGAGCGCACCA
TCTTCTTCAAGGACGACGGCAACTACAAGACCCGCGCCGAGGTGAAGTTCGAGGGCGACACCCTGGTGAACCGCATCGAGCTGAAGGGCATCGACTTCAA
GGAGGACGGCAACATCCTGGGGCACAAGCTGGAGTACAACTACAACAGCCACAACGTCTATATCATGGCCGACAAGCAGAAGAACGGCATCAAGGTGAAC
TTCAAGATCCGCCACAACATCGAGGACGGCAGCGTGCAGCTCGCCGACCACTACCAGCAGAACACCCCCATCGGCGACGGCCCCGTGCTGCTGCCCGACA
ACCACTACCTGAGCACCCAGTCCGCCCTGAGCAAAGACCCCAACGAGAAGCGCGATCACATGGTCCTGCTGGAGTTCGTGACCGCCGCCGGGATCACTCT
CGGCATGGACGAGCTGTACAAGTAAATCATTCCACTCAACATTCAGGCTCCTCTGCGCACGTAAAGTGCCAAAGGCAATACCCTGCGGCCGCCACCGCGG
TGGAGCTCGAATATTCTATGATCTGTCTTTTTATATTTGTAGCAGAGT

>P_{tub}-2，11bp deletion

CATTGAATTGACCATTCATCCTCCGTTAAACCCAAAGCAGAGCTTCGGAGAAAGGATAGGACAGAATATTTTCCATCCTGCCCCTCCAAATTTACCCCTC
TATCTGCATTGAACGACCATGTCTTCCAGATCGCAATTGAGATACTCTGAACGAGCACGAAACCATCCTAATGTGCTCGCGAAGAAGCTGTTCGAAATAG
CGGAGGCAAAGAAGACTAATGTCACTGTATCTGCCGATGTCACGACTACGAAGGAACTGCTGGATCTAGCTAACCGTAAGTTGGAACTTATTACTCTCAA
TTACACGCATCCGCGCATTCGAAGGTCAGGTTTTTTTTTAAGCTAACAATCTATCTGCCCTATACAGGTCTCGGCCCCCATATCGCCGTGATCAAAACCC
ACATTGACATCCTATCCGACTTCAGCGAAGAGACCATCTCCGGCCTGACGGCGCTCGCGAAGAAGCACAACTTCCTCATCTTTGAGGACCGCAAGTTCAT
CGATATCGGTAACACCGTCCAGAAGCAGTACCATGGTGGAGCTCTCCGGATATCTGAGTGGTCCCACATTATCAATTGCGCCATCCTCCCCGGTGAGGGT
ATCGTCGAGGCTCTCGCCCAAACTGCTTCGTCGCCAGATTTCCCATACGGAAAGGAGAGGGGACTGCTGATTCTAGCAGAGATGACCTCCAAGGGATCCC
TTGCCACGGGAGAGTACA___________GTCGACTATGCCAGGAAGTACAAGGATTTCGTCATGGGCTTTGTTTCGACGAGGGCATTGTCGGAGATCAAA
TCGGACGTGACCACGGCTTCGGATGATGAGGACTTTGTGGTTTTCACCACCGGTGTCAATCTGTCGTCGAAGGGTGATAAGCTTGGACAGCAGTACCAGA
CACCTCAGTCAGCGATCGGCCGCGGTGCGGACTTTATTATTGCTGGTCGTGGGATCTACGCTGCGCCGGATCCTGTTGAGGCTGTTCAGCGCTACCAGCA
GGAGGGTTGGGAGGCATATGAGGCTAGGGTTGGCAAGCAGTGAGAATATTCTATGATCTGTCTTTTTATATTTGTAGCAGAGTCGTGGCTATATGACCCT
TTTGTTACCCAGATACAGTATACATACCACGTGATGATTTCAACTAGGTATTTTCCAAACAGGAAATAAAGAAGCTCACTTTCTAACCCATTTCAAATCC
CAATATGCCGAATAGC

>P_{tub}-3，1583 bp HR

ATTTACCCCTCTATCTGCATTGAACGACCCCTCGAGGTCGACAGTACTGGGGCATCGACGACTTGTCACGGGAGATATTTCTGTATTTTTAGGACAAAGC
TGACGGGCATTTTATATCGAGGATCTCGGGTGACCATCCTCTCGGGGTAATTATGTTTGGGGTGGGTTAGTTAGAGCGGCTAAGTACCTACTGAACACAG
CGCCCACGAAGACCACTGGTTGATGACAAATCATAACAAATAAATACTTGACCAATTGTCGAGTATGTGCCATCAATGGGATGTCCTCAAAATGAGTATA
TTTCTGGCCATGAACGTAGTTATTAATTTCTCTGTCTCCAATGTAACCAAGAGTTCCTCTATCCAGGTCGTTCCTGCATGGACAACGCGGGCTGGTCCCG
TGACGTGGCACGAACCGGCGCGATACCGGATTGAGCGCGCCCCCTGCCCGGCCTCTTGTTGTCCTGTTGACTCCTGAACTAACCTTCTTAGCCGCACAAC
AAGGCGTCTCCTCACCGCCGACTTCTTCCCAACTTCATCCTCCATCGCTGTCCTTTCTCCCAGAACTCCTCTTGTCCTGGGTTTCAATCCTCTCCTCATC
CTCCCGTCCATTCCAACTCTCTTATTTACCGGACCTGTCACCTTCCTGCCACCATGCGTGAAATCGTACGTTTGTTGACTAGTTCTAGAGCGGCCGCATC
CACTCGTTCACTTGTTTGGTTCTTCTATTCTCACACTCGTTTTCCTCTAATGGGAATCAACATGCGTCGAGCTTTGGCCGTAGCTGCCATCGCAGCGTCC
GCCAATGCAATGGTGAGCAAGGGCGAGGAGCTGTTCACCGGGGTGGTGCCCATCCTGGTCGAGCTGGACGGCGACGTAAACGGCCACAAGTTCAGCGTGT
CCGGCGAGGGCGAGGGCGATGCCACCTACGGCAAGCTGACCCTGAAGTTCATCTGCACCACCGGCAAGCTGCCCGTGCCCTGGCCCACCCTCGTGACCAC
CCTGACCTACGGCGTGCAGTGCTTCAGCCGCTACCCCGACCACATGAAGCAGCACGACTTCTTCAAGTCCGCCATGCCCGAAGGCTACGTCCAGGAGCGC
ACCATCTTCTTCAAGGACGACGGCAACTACAAGACCCGCGCCGAGGTGAAGTTCGAGGGCGACACCCTGGTGAACCGCATCGAGCTGAAGGGCATCGACT
TCAAGGAGGACGGCAACATCCTGGGGCACAAGCTGGAGTACAACTACAACAGCCACAACGTCTATATCATGGCCGACAAGCAGAAGAACGGCATCAAGGT
GAACTTCAAGATCCGCCACAACATCGAGGACGGCAGCGTGCAGCTCGCCGACCACTACCAGCAGAACACCCCCATCGGCGACGGCCCCGTGCTGCTGCCC
GACAACCACTACCTGAGCACCCAGTCCGCCCTGAGCAAAGACCCCAACGAGAAGCGCGATCACATGGTCCTGCTGGAGTTCGTGACCGCCGCCGGGATCA
CTCTCGGCATGGACGAGCTGTACAAGTAAATCATTCCACTCAACATTCAGGCTCCTCTGCGCACGTAAAGTGCCAAAGGCAATACCCTGCGGCCGCCACC
GCGGTGGAGCTCGAATATTCTATGATCTGTCTTTTTATATTTGTAGCAGAGTCGTGGCTATATGACCCTTTTGTTACCCAGATACAGTATACATACC

>P_{tub}-4，1583 bp HR

CTCCAAATTTACCCCTCTATCTGCATTGAACGACCCCTCGAGGTCGACAGTACTGGGGCATCGACGACTTGTCACGGGAGATATTTCTGTATTTTTAGGA
CAAAGCTGACGGGCATTTTATATCGAGGATCTCGGGTGACCATCCTCTCGGGGTAATTATGTTTGGGGTGGGTTAGTTAGAGCGGCTAAGTACCTACTGA
ACACAGCGCCCACGAAGACCACTGGTTGATGACAAATCATAACAAATAAATACTTGACCAATTGTCGAGTATGTGCCATCAATGGGATGTCCTCAAAATG
AGTATATTTCTGGCCATGAACGTAGTTATTAATTTCTCTGTCTCCAATGTAACCAAGAGTTCCTCTATCCAGGTCGTTCCTGCATGGACAACGCGGGCTG
GTCCCGTGACGTGGCACGAACCGGCGCGATACCGGATTGAGCGCGCCCCCTGCCCGGCCTCTTGTTGTCCTGTTGACTCCTGAACTAACCTTCTTAGCCG
CACAACAAGGCGTCTCCTCACCGCCGACTTCTTCCCAACTTCATCCTCCATCGCTGTCCTTTCTCCCAGAACTCCTCTTGTCCTGGGTTTCAATCCTCTC
CTCATCCTCCCGTCCATTCCAACTCTCTTATTTACCGGACCTGTCACCTTCCTGCCACCATGCGTGAAATCGTACGTTTGTTGACTAGTTCTAGAGCGGC
CGCATCCACTCGTTCACTTGTTTGGTTCTTCTATTCTCACACTCGTTTTCCTCTAATGGGAATCAACATGCGTCGAGCTTTGGCCGTAGCTGCCATCGCA
GCGTCCGCCAATGCAATGGTGAGCAAGGGCGAGGAGCTGTTCACCGGGGTGGTGCCCATCCTGGTCGAGCTGGACGGCGACGTAAACGGCCACAAGTTCA
GCGTGTCCGGCGAGGGCGAGGGCGATGCCACCTACGGCAAGCTGACCCTGAAGTTCATCTGCACCACCGGCAAGCTGCCCGTGCCCTGGCCCACCCTCGT

GACCACCCTGACCTACGGCGTGCAGTGCTTCAGCCGCTACCCCGACCACATGAAGCAGCACGACTTCTTCAAGTCCGCCATGCCCGAAGGCTACGTCCAG
GAGCGCACCATCTTCTTCAAGGACGACGGCAACTACAAGACCCGCGCCGAGGTGAAGTTCGAGGGCGACACCCTGGTGAACCGCATCGAGCTGAAGGGCA
TCGACTTCAAGGAGGACGGCAACATCCTGGGGCACAAGCTGGAGTACAACTACAACAGCCACAACGTCTATATCATGGCCGACAAGCAGAAGAACGGCAT
CAAGGTGAACTTCAAGATCCGCCACAACATCGAGGACGGCAGCGTGCAGCTCGCCGACCACTACCAGCAGAACACCCCCATCGGCGACGGCCCCGTGCTG
CTGCCCGACAACCACTACCTGAGCACCCAGTCCGCCCTGAGCAAAGACCCCAACGAGAAGCGCGATCACATGGTCCTGCTGGAGTTCGTGACCGCCGCCG
GGATCACTCTCGGCATGGACGAGCTGTACAAGTAAATCATTCCACTCAACATTCAGGCTCCTCTGCGCACGTAAAGTGCCAAAGGCAATACCCTGCGGCC
GCCACCGCGGTGGAGCTCGAATATTCTATGATCTGTCTTTTTATATTTGTAGCAGAGTCGTGGCTATATGACCCTTTTGTTACCCAGATACAGTATACAT
ACCACG

>P_{tub}-5, 1 bp insertion

CCGTTAAACCCAAAGCAGAGCTTCGGAGAAAGGATAGGACAGAATATTTTCCATCCTGCCCCTCCAAATTTACCCCTCTATCTGCATTGAACGACCATGT
CTTCCAGATCGCAATTGAGATACTCTGAACGAGCACGAAACCATCCTAATGTGCTCGCGAAGAAGCTGTTCGAAATAGCGGAGGCAAAGAAGACTAATGT
CACTGTATCTGCCGATGTCACGACTACGAAGGAACTGCTGGATCTAGCTAACCGTAAGTTGGAACTTATTACTCTCAATTACACGCATCCGCGCATTCGA
AGGTCAGGTTTTTTTTAAGCTAACAATCTATCTGCCCTATACAGGTCTCGGCCCCCATATCGCCGTGATCAAAACCCACATTGACATCCTATCCGACTT
CAGCGAAGAGACCATCTCCGGCCTGACGGCGCTCGCGAAGAAGCACAACTTCCTCATCTTTGAGGACCGCAAGTTCATCGATATCGGTAACACCGTCCAG
AAGCAGTACCATGGTGGAGCTCTCCGGATATCTGAGTGGTCCCACATTATCAATTGCGCCATCCTCCCCGGTGAGGGTATCGTCGAGGCTCTCGCCCAAA
CTGCTTCGTCGCCAGATTTCCCATACGGAAAGGAGAGGGGACTGCTGATTCTAGCAGAGATGACCTCCAAGGGATCCCTTGCCACGGGAGAGTACAACGA
AGGTCTCAGTCGACTATGCCAGGAAGTACAAGGATTTCGTCATGGGCTTTGTTTCGACGAGGGCATTGTCGGAGATCAAATCGGACGTGACCACGGCTTC
GGATGATGAGGACTTTGTGGTTTTCACCACCGGTGTCAATCTGTCGTCGAAGGGTGATAAGCTTGGACAGCAGTACCAGACACCTCAGTCAGCGATCGGC
CGCGGTGCGGACTTTATTATTGCTGGTCGTGGGATCTACGCTGCGCCGGATCCTGTTGAGGCTGTTCAGCGCTACCAGCAGGAGGGTTGGGAGGCATATG
AGGCTAGGGTTGGCAAGCAGTGAGAATATTCTATGATCTGTCTTTTTATATTTGTAGCAGAGTCGTGGCTATATGACCCTTTTGTTACCCAGATACAGTA
TACATACCACGTGATGATTTCAACTAGGTATTTTCCAAACAGGAAATAAAGAAGCTCACTTTCTAACCCATTTCAAATCCCAATAT

>P_{tub}-6, 1583 bp HR

CTCCAAATTTACCCCTCTATCTGCATTGAACGACCCCTCGAGGTCGACAGTACTGGGGCATCGACGACTTGTCACGGGAGATATTTCTGTATTTTTAGGA
CAAAGCTGACGGGCATTTTATATCGAGGATCTCGGGTGACCATCCTCTCGGGGTAATTATGTTTGGGGTGGGTTAGTTAGAGCGGCTAAGTACCTACTGA
ACACAGCGCCCACGAAGACCACTGGTTGATGACAAATCATAACAAATAAATACTTGACCAATTGTCGAGTATGTGCCATCAATGGGATGTCCTCAAAATG
AGTATATTTCTGGCCATGAACGTAGTTATTAATTTCTCTGTCTCCAATGTAACCAAGAGTTCCTCTATCCAGGTCGTTCCTGCATGGACAACGCGGGCTG
GTCCCGTGACGTGGCACGAACCGGCGCGATACCGGATTGAGCGCGCCCCCTGCCCGGCCTCTTGTTGTCCTGTTGACTCCTGAACTAACCTTCTTAGCCG
CACAACAAGGCGTCTCCTCACCGCCGACTTCTTCCCAACTTCATCCTCCATCGCTGTCCTTTCTCCCAGAACTCCTCTTGTCCTGGGTTTCAATCCTCTC
CTCATCCTCCCGTCCATTCCAACTCTCTTATTTACCGGACCTGTCACCTTCCTGCCACCATGCGTGAAATCGTACGTTTGTTGACTAGTTCTAGAGCGGC
CGCATCCACTCGTTCACTTGTTTGGTTCTTCTATTCTCACACTCGTTTTCCTCTAATGGGAATCAACATGCGTCGAGCTTTGGCCGTAGCTGCCATCGCA
GCGTCCGCCAATGCAATGGTGAGCAAGGGCGAGGAGCTGTTCACCGGGGTGGTGCCCATCCTGGTCGAGCTGGACGGCGACGTAAACGGCCACAAGTTCA
GCGTGTCCGGCGAGGGCGAGGGCGATGCCACCTACGGCAAGCTGACCCTGAAGTTCATCTGCACCACCGGCAAGCTGCCCGTGCCCTGGCCCACCCTCGT
GACCACCCTGACCTACGGCGTGCAGTGCTTCAGCCGCTACCCCGACCACATGAAGCAGCACGACTTCTTCAAGTCCGCCATGCCCGAAGGCTACGTCCAG
GAGCGCACCATCTTCTTCAAGGACGACGGCAACTACAAGACCCGCGCCGAGGTGAAGTTCGAGGGCGACACCCTGGTGAACCGCATCGAGCTGAAGGGCA
TCGACTTCAAGGAGGACGGCAACATCCTGGGGCACAAGCTGGAGTACAACTACAACAGCCACAACGTCTATATCATGGCCGACAAGCAGAAGAACGGCAT
CAAGGTGAACTTCAAGATCCGCCACAACATCGAGGACGGCAGCGTGCAGCTCGCCGACCACTACCAGCAGAACACCCCCATCGGCGACGGCCCCGTGCTG
CTGCCCGACAACCACTACCTGAGCACCCAGTCCGCCCTGAGCAAAGACCCCAACGAGAAGCGCGATCACATGGTCCTGCTGGAGTTCGTGACCGCCGCCG
GGATCACTCTCGGCATGGACGAGCTGTACAAGTAAATCATTCCACTCAACATTCAGGCTCCTCTGCGCACGTAAAGTGCCAAAGGCAATACCCTGCGGCC
GCCACCGCGGTGGAGCTCGAATATTCTATGATCTGTCTTTTTATATTTGTAGCAGAGTCGTGGCTATATGACCCTTTTGTTACCCAGATACAGTATACAT
ACCAC

>P_{tef1}-1, 73 bp insertion

CTGCCCCTCCAAATTTACCCCTCTATCTGCATTGAACGACCATGTCTTCCAGATCGCAATTGAGATACTCTGAACGAGCACGAAACCATCCTAATGTGCT
CGCGAAGAAGCTGTTCGAAATAGCGGAGGCAAAGAAGACTAATGTCACTGTATCTGCCGATGTCACGACTACGAAGGAACTGCTGGATCTAGCTAACCGT
AAGTTGGAACTTATTACTCTCAATTACACGCATCCGCGCATTCGAAGGTCAGGTTTTTTTTAAGCTAACAATCTATCTGCCCTATACAGGTCTCGGCCC
CCATATCGCCGTGATCAAAACCCACATTGACATCCTATCCGACTTCAGCGAAGAGACCATCTCCGGCCTGACGGCGCTCGCGAAGAAGCACAACTTCCTC
ATCTTTGAGGACCGCAAGTTCATCGATATCGGTAACACCGTCCAGAAGCAGTACCATGGTGGAGCTCTCCGGATATCTGAGTGGTCCCACATTATCAATT
GCGCCATCCTCCCCGGTGAGGGTATCGTCGAGGCTCTCGCCCAAACTGCTTCGTCGCCAGATTTCCCATACGGAAAGGAGAGGGGACTGCTGATTCTAGC
AGAGATGACCTCCAAGGGATCCCTTGCCACGGGAGAGTACAATCCTGGTCGAGCTGGACGGCGACGTAAACGGCCACAAGTTCAGCGTGTCCGGCGAGGG
CGAGGGCGATGCCACGAAGGTCTCAGTCGACTATGCCAGGAAGTACAAGGATTTCGTCATGGGCTTTGTTTCGACGAGGGCATTGTCGGAGATCAAATCG
GACGTGACCACGGCTTCGGATGATGAGGACTTTGTGGTTTTCACCACCGGTGTCAATCTGTCGTCGAAGGGTGATAAGCTTGGACAGCAGTACCAGACAC

CTCAGTCAGCGATCGGCCGCGGTGCGGACTTTATTATTGCTGGTCGTGGGATCTACGCTGCGCCGGATCCTGTTGAGGCTGTTCAGCGCTACCAGCAGGA
GGGTTGGGAGGCATATGAGGCTAGGGTTGGCAAGCAGTGAGAATATTCTATGATCTGTCTTTTTATATTTGTAGCAGAGTCGTGGCTATATGACCCTTTT
GTTACCCAGATACAGTATACATACCACGTGATGATTT

>P_{tef1}-2, 106 bp insertion

GCCCCTCCAAATTTACCCCTCTATCTGCATTGAACGACCATGTCTTCCAGATCGCAATTGAGATACTCTGAACGAGCACGAAACCATCCTAATGTGCTCG
CGAAGAAGCTGTTCGAAATAGCGGAGGCAAAGAAGACTAATGTCACTGTATCTGCCGATGTCACGACTACGAAGGAACTGCTGGATCTAGCTAACCGTAA
GTTGGAACTTATTACTCTCAATTACACGCATCCGCGCATTCGAAGGTCAGGTTTTTTTTTAAGCTAACAATCTATCTGCCCTATACAGGTCTCGGCCCCC
ATATCGCCGTGATCAAAACCCACATTGACATCCTATCCGACTTCAGCGAAGAGACCATCTCCGGCCTGACGGCGCTCGCGAAGAAGCACAACTTCCTCAT
CTTTGAGGACCGCAAGTTCATCGATATCGGTAACACCGTCCAGAAGCAGTACCATGGTGGAGCTCTCCGGATATCTGAGTGGTCCCACATTATCAATTGC
GCCATCCTCCCCGGTGAGGGTATCGTCGAGGCTCTCGCCCAAACTGCTTCGTCGCCAGATTTCCCATACGGAAAGGAGAGGGGACTGCTGATTCTAGCAG
AGATGACCTCCAAGGGATCCCTTGCCACGGGAGAGTACAAGCACCGACTCGGTGCCACTTTTTCAAGTTGATAACGGACTAGCCTTATTTTAACTTGCTA
TTTCTAGCTCTAAAACTCGTGTACTCTCCCGTGGCACCTATAGTACGAAGGTCTCAGTCGACTATGCCAGGAAGTACAAGGATTTCGTCATGGGCTTTGT
TTCGACGAGGGCATTGTCGGAGATCAAATCGGACGTGACCACGGCTTCGGATGATGAGGACTTTGTGGTTTTCACCACCGGTGTCAATCTGTCGTCGAAG
GGTGATAAGCTTGGACAGCAGTACCAGACACCTCAGTCAGCGATCGGCCGCGGTGCGGACTTTATTATTGCTGGTCGTGGGATCTACGCTGCGCCGGATC
CTGTTGAGGCTGTTCAGCGCTACCAGCAGGAGGGTTGGGAGGCATATGAGGCTAGGGTTGGCAAGCAGTGAGATATTCTATGATCTG

>P_{tef1}-3, 1941 bp HR

TTACCCCTCTATCTGCATTGAACGACCCCTCGAGGTCGACCACAGCTACGTATCGTATTGACATCAAAGGAAAATTGTACAATTATTATTCCCCAATATA
CCTGCAGCCACCCGGTATCTGGATCTAGATCTTGAACTGTAACCTTGTCCTTTCCCGAAGACGGGATGTGGCAACAAAGAAGAAAAATTCTTCGTCGAG
GCTCCCACGGCGCCAATTCCAAAATAAAAGGAAGCTTGATGGATATGTCAATCACAGCCCCGCATCAAGCTCTTTTTGGCGACCGCGCCACTTGTTCGCT
GGCGACGGATGGCAAATGGGAGGAGGGGCATCTGGTAAAAGCATGCAAACATGAGGGATAGCGCTTGCATCCAGGCCAGCGCAGCGGTGTGGGCTGCTGG
CGTTGATGCCCAGTACACTGCATCGGGACCCGTTATTTGAGGGGAGGTTGATGGGCAGGAGAGTCAAGGAGAGTCCAAGGAGAGTCAGAAGAGTGGGGTC
CGCAGCGAAAACTTCTACCAGGGTTCGCCGCTGGCTGGGCAGGCAGTCTTTTCTTGCCGCCAAAACAGCTGAGCTCTTTGGTCGAGTCCGCCAGCCGACA
GTTACTACTAAGTGAAGATTCTGCGCTGGTGCCGCACAACCAGGGTGGGGCATCGTCCCCTCCACGGCCAATGGCACGCACGGCTGCGGGAGGGCTTGAG
CCCGCCTGCTTAGTCGCCCTCTCGCTTGCCCCTCCATATAAGGATTTCCCCTCCTCCCCTCCCACAAGATTTTTTCCTTCTTTCTCCCCCTCGTCTGCTT
CAGTACGTATAACTCCCCTCCCGTCCAGCTTCGCGCTTCCTCTTCCTTTCTTCTCACTAACCATCTGTCTCAGCACCTCTACGCATTCGGCCGTAGTATC
TGAGCGCCCTCCGGAATCCTTCTAGTATCTTACAAAGACACTACAAGAATTCACATCTCACAACACCGCCGCCATGGGGTAAGCAGGACCTCGATTCCAT
CAAAAAAATCCATCGAGGATGGGCTTCATTCGGACTAGTTCTAGAGCGGCCGCATCCACTCGTTCACTTGTTTGGTTCTTCTATTCTCACACTCGTTTTC
CTCTAATGGGAATCAACATGCGTCGAGCTTTGGCCGTAGCTGCCATCGCAGCGTCCGCCAATGCAATGGTGAGCAAGGGCGAGGAGCTGTTCACCGGGGT
GGTGCCCATCCTGGTCGAGCTGGACGGCGACGTAAACGGCCACAAGTTCAGCGTGTCCGGCGAGGGCGAGGGCGATGCCACCTACGGCAAGCTGACCCTG
AAGTTCATCTGCACCACCGGCAAGCTGCCCGTGCCCTGGCCCACCCTCGTGACCACCCTGACCTACGGCGTGCAGTGCTTCAGCCGCTACCCCGACCACA
TGAAGCAGCACGACTTCTTCAAGTCCGCCATGCCCGAAGGCTACGTCCAGGAGCGCACCATCTTCTTCAAGGACGACGGCAACTACAAGACCCGCGCCGA
GGTGAAGTTCGAGGGCGACACCCTGGTGAACCGCATCGAGCTGAAGGGCATCGACTTCAAGGAGGACGGCAACATCCTGGGGCACAAGCTGGAGTACAAC
TACAACAGCCACAACGTCTATATCATGGCCGACAAGCAGAAGAACGGCATCAAGGTGAACTTCAAGATCCGCCACAACATCGAGGACGGCAGCGTGCAGC
TCGCCGACCACTACCAGCAGAACACCCCCATCGGCGACGGCCCCGTGCTGCTGCCCGACAACCACTACCTGAGCACCCAGTCCGCCCTGAGCAAAGACCC
CAACGAGAAGCGCGATCACATGGTCCTGCTGGAGTTCGTGACCGCCGCCGGGATCACTCTCGGCATGGACGAGCTGTACAAGTAAATCATTCCACTCAAC
ATTCAGGCTCCTCTGCGCACGTAAAGTGCCAAAGGCAATACCCTGCGGCCGCCACCGCGGTGGAGCTCGAATATTCTATGATCTGTCTTTTTATATTTGT
AGCAGAGTCGTGGCTATATGACCCTTTTGTTACCCAGATACAGTATACATACCA

>P_{gpdA}-1, 1960 HR

ATCCTGCCCCTCCAAATTTACCCCTCTATCTGCATTGAACGACCCCTCGAGGTCGACCTAGAAATTCCGGCTAAAGGTGCTTTGGCTAAACATAAGTTGG
TAAGTGTCTGGGTGAAGGGAGATCCAATGAGAAACATCGTTTCTCTATTTTACAGACGGTAAATATAGCAGACCGATTGATTACCTATCGCTTGAACATG
CAGCCGTCAGTTGTCCTCATGGGCCTAAAAGACCCCGCCTCTAATACACTCTTCTAATCCATCGTAGAGTAATGCATGTACCTGGTAGATCATAATAAAA
ATGCCTACCATACCTCACAAGGTGCTCGAGCTGTAGGCAAAGTCCGAAGCGCTATTTCCCATAGATCCTTTATGCATGGCAAAACGCCGCAAACTAGACT
GGACCCTTGCAATGTCAAGGTCTAGACAAAATAAGCGCCTGAGACCCTTGCGGTTGTCCATCTCCGCACATGCAGCGGCCGGGACCGAACTGTCAGATTC
GATTTATTTACAGGATGCGATCGATAAGTCTATTGCGTCCACACGCTGTCACTCCAAATAGGACCGGTTAGAACGATCAAACAAGACCGTAGAATAAACA
CGAAAGCACACACGACGTGTCTCGGGCCTAAATCTGTTGGGATTGTCAGACCACAAGGATGGGATCCATGGCCCCCCAGAAAGTTGCAATGGCTGCTGT
CTTTTTAGCCACCACCACGACCCTTGCCATCGATGTTGGGTGATCATATCACCCTCCTGAATAATGGGACGACGGATGCAGCAACCAGTGAATGTATCGT
ATCACGTTGGGTAGTTTCATCGACGAGGACTTATATAGATAATGCCAGTACCTGTCTTCGTAGGTCTGCTCATGCACCTTGCGTCTCTCATATAGTTTGC
AGACACAGATCTCTATCCACTGTTTCAGTGGTCCTTGGCTTCTTGAATACTTTTCCCCGACTCCTTACCGTGACAATACGTGACGAAACCCCTCGCCGTC
TTTAGCCGGTGTCAATTCAAACAAAATGGTTGCAAAGGTAAGCGACCGACGAGTTGTTGATGCGAATCCACTAGTTCTAGAGCGGCCGCATCCACTCGTT
CACTTGTTTGGTTCTTCTATTCTCACACTCGTTTTCCTCTAATGGGAATCAACATGCGTCGAGCTTTGGCCGTAGCTGCCATCGCAGCGTCCGCCAATGC

AATGGTGAGCAAGGGCGAGGAGCTGTTCACCGGGGTGGTGCCCATCCTGGTCGAGCTGGACGGCGACGTAAACGGCCACAAGTTCAGCGTGTCCGGCGAG
GGCGAGGGCGATGCCACCTACGGCAAGCTGACCCTGAAGTTCATCTGCACCACCGGCAAGCTGCCCGTGCCCTGGCCCACCCTCGTGACCACCCTGACCT
ACGGCGTGCAGTGCTTCAGCCGCTACCCCGACCACATGAAGCAGCACGACTTCTTCAAGTCCGCCATGCCCGAAGGCTACGTCCAGGAGCGCACCATCTT
CTTCAAGGACGACGGCAACTACAAGACCCGCGCCGAGGTGAAGTTCGAGGGCGACACCCTGGTGAACCGCATCGAGCTGAAGGGCATCGACTTCAAGGAG
GACGGCAACATCCTGGGGCACAAGCTGGAGTACAACTACAACAGCCACAACGTCTATATCATGGCCGACAAGCAGAAGAACGGCATCAAGGTGAACTTCA
AGATCCGCCACAACATCGAGGACGGCAGCGTGCAGCTCGCCGACCACTACCAGCAGAACACCCCCATCGGCGACGGCCCCGTGCTGCTGCCCGACAACCA
CTACCTGAGCACCCAGTCCGCCCTGAGCAAAGACCCCAACGAGAAGCGCGATCACATGGTCCTGCTGGAGTTCGTGACCGCCGCCGGGATCACTCTCGGC
ATGGACGAGCTGTACAAG**TAA**ATCATTCCACTCAACATTCAGGCTCCTCTGCGCACGTAAAGTGCCAAAGGCAATACCCTGCGGCCGCCACCGCGGTGGA
GCTCGAATATTCTATGATCTGTCTTTTTATATTTGTAGCAGAGTCGTGGCTATATGACCCTTTTGTTACCCAGATACAGTATACATACCACGTGA

>P_{gpdA}-2’ , >327 bp insertion

TGCCCCTCCAAATTTACCCCTCTATCTGCATTGAACGACCATGTCTTCCAGATCGCAATTGAGATACTCTGAACGAGCACGAAACCATCCTAATGTGCTC
GCGAAGAAGCTGTTCGAAATAGCGGAGGCAAAGAAGACTAATGTCACTGTATCTGCCGATGTCACGACTACGAAGGAACTGCTGGATCTAGCTAACCGTA
AGTTGGAACTTATTACTCTCAATTACACGCATCCGCGCATTCGAAGGTCAGGTTTTTTTTTAAGCTAACAATCTATCTGCCCTATACAGGTCTCGGCCCC
CATATCGCCGTGATCAAAACCCACATTGACATCCTATCCGACTTCAGCGAAGAGACCATCTCCGGCCTGACGGCGCTCGCGAAGAAGCACAACTTCCTCA
TCTTTGAGGACCGCAAGTTCATCGATATCGGTAACACCGTCCAGAAGCAGTACCATGGTGGAGCTCTCCGGATATCTGAGTGGTCCCACATTATCAATTG
CGCCATCCTCCCCGGTGAGGGTATCGTCGAGGCTCTCGCCCAAACTGCTTCGTCGCCAGATTTCCCATACGGAAAGGAGAGGGGACTGCTGATTCTAGCA
GAGATGACCTCCAAGGGATCCCTTGCCACGGAACGGCATCAAGGTGAACTTCAAGATCCGCCACAACATCGAGGACGGCAGCGTGCAGCTCGCCGACCAC
TACCAGCAGAACACCCCCATCGGCGACGGCCCCGTGCTGCTGCCCGACAACCACTACCTGAGCACCCAGTCCGCCCTGAGCAAAGACCCCAACGAGAAGC
GCGATCACATGGTCCTGCTGGAGTTCGTGACCGCCGCCGGGATCACTCTCGGCATGGACGAGCTGTACAAGTAAATCATTCCACTCAACATTCAGGCTCC
TCTGCGCACGTAAAGTGCCAAAGGCAATACCCTGCGGCCGCCACCGCGGTGGAGCTCGAATATTCTATGATCTGTCTTTTTATATTTGTAGCAGAGTCGT
GGCTATATGACCCTTTTGTTACCCAGATACAGTATACATAC

>P_{gpdA}-3, 8 bp deletion

GCCCCTCCAAATTTACCCCTCTATCTGCATTGAACGACCATGTCTTCCAGATCGCAATTGAGATACTCTGAACGAGCACGAAACCATCCTAATGTGCTCG
CGAAGAAGCTGTTCGAAATAGCGGAGGCAAAGAAGACTAATGTCACTGTATCTGCCGATGTCACGACTACGAAGGAACTGCTGGATCTAGCTAACCGTAA
GTTGGAACTTATTACTCTCAATTACACGCATCCGCGCATTCGAAGGTCAGGTTTTTTTTTAAGCTAACAATCTATCTGCCCTATACAGGTCTCGGCCCCC
ATATCGCCGTGATCAAAACCCACATTGACATCCTATCCGACTTCAGCGAAGAGACCATCTCCGGCCTGACGGCGCTCGCGAAGAAGCACAACTTCCTCAT
CTTTGAGGACCGCAAGTTCATCGATATCGGTAACACCGTCCAGAAGCAGTACCATGGTGGAGCTCTCCGGATATCTGAGTGGTCCCACATTATCAATTGC
GCCATCCTCCCCGGTGAGGGTATCGTCGAGGCTCTCGCCCAAACTGCTTCGTCGCCAGATTTCCCATACGGAAAGGAGAGGGGACTGCTGATTCTAGCAG
AGATGACCTCCAAGGGATCCCTTGCCACGGGAGAGTACA__________TCAGTCGACTATGCCAGGAAGTACAAGGATTTCGTCATGGGCTTTGTTTCG
ACGAGGGCATTGTCGGAGATCAAATCGGACGTGACCACGGCTTCGGATGATGAGGACTTTGTGGTTTTCACCACCGGTGTCAATCTGTCGTCGAAGGGTG
ATAAGCTTGGACAGCAGTACCAGACACCTCAGTCAGCGATCGGCCGCGGTGCGGACTTTATTATTGCTGGTCGTGGGATCTACGCTGCGCCGGATCCTGT
TGAGGCTGTTCAGCGCTACCAGCAGGAGGGTTGGGAGGCATATGAGGCTAGGGTTGGCAAGCAGTGAGAATATTCTATGATCTGTCTTTTTATATTTGTA
GCAGAGTCGTGGCTATATGACCCTTTTGTTACCCAGATACAGTATACA

>P_{gpdA}-4, 1960 bp HR

TTTACCCCTCTATCTGCATTGAACGACCCCTCGAGGTCGACCTAGAAATTCCGGCTAAAGGTGCTTTGGCTAAACATAAGTTGGTAAGTGTCTGGGTGAA
GGGAGATCCAATGAGAAACATCGTTTCTCTATTTTACAGACGGTAAATATAGCAGACCGATTGATTACCTATCGCTTGAACATGCAGCCGTCAGTTGTCC
TCATGGGCCTAAAAGACCCCGCCTCTAATACACTCTTCTAATCCATCGTAGAGTAATGCATGTACCTGGTAGATCATAATAAAAATGCCTACCATACCTC
ACAAGGTGCTCGAGCTGTAGGCAAAGTCCGAAGCGCTATTTCCCATAGATCCTTTATGCATGGCAAAACGCCGCAAACTAGACTGGACCCTTGCAATGTC
AAGGTCTAGACAAAATAAGCGCCTGAGACCCTTGCGGTTGTCCATCTCCGCACATGCAGCGGCCGGGACCGAACTGTCAGATTCGATTTATTTACAGGAT
GCGATCGATAAGTCTATTGCGTCCACACGCTGTCACTCCAAATAGGACCGGTTAGAACGATCAAACAAGACCGTAGAATAAACACGAAAGCACACACGAC
GTGTCTCGGGCCTAAATCTGTTGGGATTGTCAGACCACAAGGATGGGATCCATGGCCCCCCCAGAAAGTTGCAATGGCTGCTGTCTTTTTAGCCACCACC
ACGACCCTTGCCATCGATGTTGGGTGATCATATCACCCTCCTGAATAATGGGACGACGGATGCAGCAACCAGTGAATGTATCGTATCACGTTGGGTAGTT
TCATCGACGAGGACTTATATAGATAATGCCAGTACCTGTCTTCGTAGGTCTGCTCATGCACCTTGCGTCTCTCATATAGTTTGCAGACACAGATCTCTAT
CCACTGTTTCAGTGGTCCTTGGCTTCTTGAATACTTTTCCCCGACTCCTTACCGTGACAATACGTGACGAAACCCCTCGCCGTCTTTAGCCGGTGTCAAT
TCAAACAAAATGGTTGCAAAGGTAAGCGACCGACGAGTTGTTGATGCGAATCCACTAGTTCTAGAGCGGCCGCATCCACTCGTTCACTTGTTTGGTTCTT
CTATTCTCACACTCGTTTTCCTCTAATGGGAATCAACATGCGTCGAGCTTTGGCCGTAGCTGCCATCGCAGCGTCCGCCAATGCAATGGTGAGCAAGGGC
GAGGAGCTGTTCACCGGGGTGGTGCCCATCCTGGTCGAGCTGGACGGCGACGTAAACGGCCACAAGTTCAGCGTGTCCGGCGAGGGCGAGGGCGATGCCA
CCTACGGCAAGCTGACCCTGAAGTTCATCTGCACCACCGGCAAGCTGCCCGTGCCCTGGCCCACCCTCGTGACCACCCTGACCTACGGCGTGCAGTGCTT
CAGCCGCTACCCCGACCACATGAAGCAGCACGACTTCTTCAAGTCCGCCATGCCCGAAGGCTACGTCCAGGAGCGCACCATCTTCTTCAAGGACGACGGC
AACTACAAGACCCGCGCCGAGGTGAAGTTCGAGGGCGACACCCTGGTGAACCGCATCGAGCTGAAGGGCATCGACTTCAAGGAGGACGGCAACATCCTGG

GGCACAAGCTGGAGTACAACTACAACAGCCACAACGTCTATATCATGGCCGACAAGCAGAAGAACGGCATCAAGGTGAACTTCAAGATCCGCCACAACAT
CGAGGACGGCAGCGTGCAGCTCGCCGACCACTACCAGCAGAACACCCCCATCGGCGACGGCCCCGTGCTGCTGCCCGACAACCACTACCTGAGCACCCAG
TCCGCCCTGAGCAAAGACCCCAACGAGAAGCGCGATCACATGGTCCTGCTGGAGTTCGTGACCGCCGCCGGGATCACTCTCGGCATGGACGAGCTGTACA
AGTAAATCATTCCACTCAACATTCAGGCTCCTCTGCGCACGTAAAGTGCCAAAGGCAATACCCTGCGGCCGCCACCGCGGTGGAGCTCGAATATTCTATG
ATCTGTCTTTTTATATTTGTAGCAGAGTCGTGGCTATATGACCCTTTTGTTACCCAGATACAGTATACATACCACGTGA

>P_{gpdA}-5, 1960 bp HR

ATCCTGCCCCTCCAAATTTACCCCTCTATCTGCATTGAACGACCCCTCGAGGTCGACCTAGAAATTCCGGCTAAAGTGTGCTTTGGCTAAACATAAGTTG
GTAAGTGTCTGGGTGAAGGGAGATCCAATGAGAAACATCGTTTCTCTATTTTACAGACGGTAAATATAGCAGACCGATTGATTACCTATCGCTTGAACAT
GCAGCCGTCAGTTGTCCTCATGGGCCTAAAAGACCCCGCCTCTAATACACTCTTCTAATCCATCGTAGAGTAATGCATGTACCTGGTAGATCATAATAAA
AATGCCTACCATACCTCACAAGGTGCTCGAGCTGTAGGCAAAGTCCGAAGCGCTATTTCCCATAGATCCTTTATGCATGGCAAAACGCCGCAAACTAGAC
TGGACCCTTGCAATGTCAAGGTCTAGACAAAATAAGCGCCTGAGACCCTTGCGGTTGTCCATCTCCGCACATGCAGCGGCCGGGACCGAACTGTCAGATT
CGATTTATTTACAGGATGCGATCGATAAGTCTATTGCGTCCACACGCTGTCACTCCAAATAGGACCGGTTAGAACGATCAAACAAGACCGTAGAATAAAC
ACGAAAGCACACACGACGTGTCTCGGGCCTAAATCTGTTGGGATTGTCAGACCACAAGGATGGGATCCATGGCCCCCCCAGAAAGTTGCAATGGCTGCTG
TCTTTTTAGCCACCACCACGACCCTTGCCATCGATGTTGGGTGATCATATCACCCTCCTGAATAATGGGACGACGGATGCAGCAACCAGTGAATGTATCG
TATCACGTTGGGTAGTTTCATCGACGAGGACTTATATAGATAATGCCAGTACCTGTCTTCGTAAGTCTGCTCATGCACCTTGCGTCTCTCATATAGTTTG
CAGACACAGATCTCTATCCACTGTTTCAGTGGTCCTTGGCTTCTTGAATACTTTTCCCCGACTCCTTACCGTGACAATACGTGACGAAACCCCTCGCCGT
CTTTAGCCGGTGTCAATTCAAACAAAATGGTTGCAAAGGTAAGCGACCGACGAGTTGTTGATGCGAATCCACTAGTTCTAGAGCGGCCGCATCCACTCGT
TCACTTGTTTGGTTCTTCTATTCTCACACTCGTTTTCCTCTAATGGGAATCAACATGCGTCGAGCTTTGGCCGTAGCTGCCATCGCAGCGTCCGCCAATG
CAATGGTGAGCAAGGGCGAGGAGCTGTTCACCGGGGTGGTGCCCATCCTGGTCGAGCTGGACGGCGACGTAAACGGCCACAAGTTCAGCGTGTCCGGCGA
GGGCGAGGGCGATGCCACCTACGGCAAGCTGACCCTGAAGTTCATCTGCACCACCGGCAAGCTGCCCGTGCCCTGGCCCACCCTCGTGACCACCCTGACC
TACGGCGTGCAGTGCTTCAGCCGCTACCCCGACCACATGAAGCAGCACGACTTCTTCAAGTCCGCCATGCCCGAAGGCTACGTCCAGGAGCGCACCATCT
TCTTCAAGGACGACGGCAACTACAAGACCCGCGCCGAGGTGAAGTTCGAGGGCGACACCCTGGTGAACCGCATCGAGCTGAAGGGCATCGACTTCAAGGA
GGACGGCAACATCCTGGGGCACAAGCTGGAGTACAACTACAACAGCCACAACGTCTATATCATGGCCGACAAGCAGAAGAACGGCATCAAGGTGAACTTC
AAGATCCGCCACAACATCGAGGACGGCAGCGTGCAGCTCGCCGACCACTACCAGCAGAACACCCCCATCGGCGACGGCCCCGTGCTGCTGCCCGACAACC
ACTACCTGAGCACCCAGTCCGCCCTGAGCAAAGACCCCAACGAGAAGCGCGATCACATGGTCCTGCTGGAGTTCGTGACCGCCGCCGGGATCACTCTCGG
CATGGACGAGCTGTACAAGTAAATCATTCCACTCAACATTCAGGCTCCTCTGCGCACGTAAAGTGCCAAAGGCAATACCCTGCGGCCGCCACCGCGGTGG
AGCTCGAATATTCTATGATCTGTCTTTTTATATTTGTAGCAGAGTCGTGGCTATATGACCCTTTTGTTACCCAGATACAGTATACATACCACGTG
