# Supplementary material for: Development of a CRISPR/Cas9 RNP-mediated genetic engineering system in Paecilomyces variotii
Source: J Microbiol. 2025 Jun 30;63(6):e2502011. doi: 10.71150/jm.2502011 (PMC13577017; doi:10.71150/jm.2502011)
Supplement: Supplementary file 2 [file jm-2502011-Supplementary-Fig-S1.pdf]

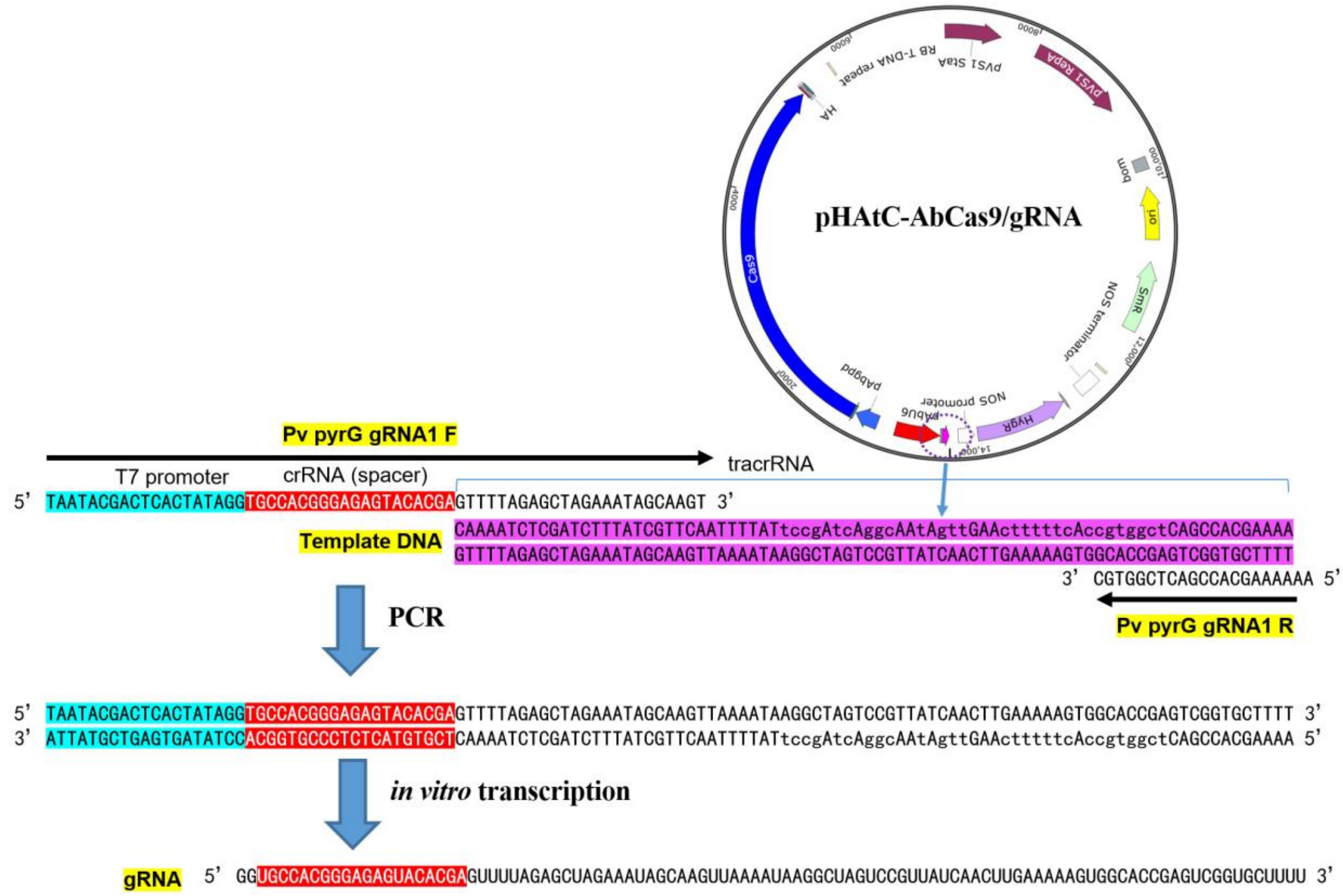

Fig. S1. Generation of gDNA. The DNA fragment consisted of T7 promoter-crRNA-tracrRNA was PCR-amplified using the primer set 1 (Pv pyrG gRNA1 F and Pv pyrG gRNA1 R). The resulting PCR products were subjected to in vitro transcription to produce gRNA.
