# Supplementary material for: Development of a CRISPR/Cas9 RNP-mediated genetic engineering system in Paecilomyces variotii
Source: J Microbiol. 2025 Jun 30;63(6):e2502011. doi: 10.71150/jm.2502011 (PMC13577017; doi:10.71150/jm.2502011)
Supplement: Supplementary file 3 [file jm-2502011-Supplementary-Fig-S2.pdf]

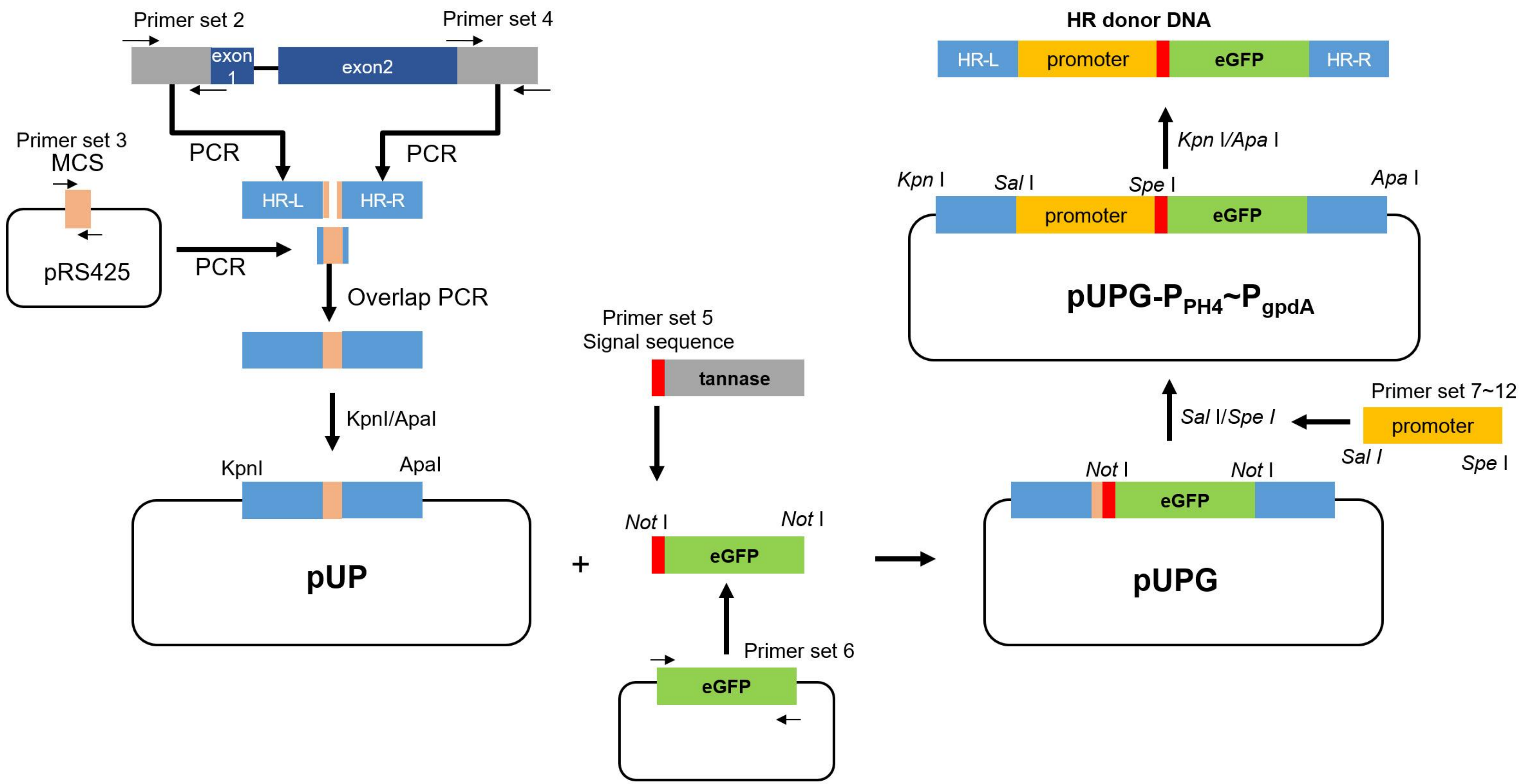

Fig. S2. Construction of HR donor DNA fragments. The HR-L and HR-R regions of *pyrG*, separated by multicloning site (MCS) were inserted to the pTOP vector to make pUP. The eGFP having a tannase signal sequence was amplified and inserted into the *Not*I site of pUP to generate pUPG. The six constitutive promoter sequences were amplified from the chromosomal DNA of P. variotii and were inserted into the *Sal*I and *Spe*I sites of pUPG to make pUPG-P. The HR donor DNA were generated by *Kpn*I/*Apa*I digestion of the pUPG-P plasmids.
