# Supplementary material for: Development of a CRISPR/Cas9 RNP-mediated genetic engineering system in Paecilomyces variotii
Source: J Microbiol. 2025 Jun 30;63(6):e2502011. doi: 10.71150/jm.2502011 (PMC13577017; doi:10.71150/jm.2502011)
Supplement: Supplementary file 4 [file jm-2502011-Supplementary-Table-S1.pdf]

Table S1. Primers used in this study

Module name	Target sequence	Primer set number	Primer name	Nucleotide sequence (5' to 3')[a]
pyrG		1	Pv pyrG gRNA1 F	TAATACGACTCACTATAGGTGCCACGGGAGAGTACACGAGTTTTAGAGCTAGAAATAGCAAGT
			Pv pyrG gRNA1 R	AAAAAAGCACCGACTCGGTGC
HR-L-MCS-HR-R	HR-L	2	Pv pyrG 5'flk F(KpnI)	ATGCATggtacCAGCTTCCAGACCTTCAGG
			Pv pyrG 5'flk R(MCS)	CGACCTCGAGGGGTCGTTCAATGCAGATAGAG
	MCS	3	MCS F(pyrG 5'flk)	CATTGAACGACCCCTCGAGGTCGACGGTATC
			MCS R(pyrG 3'flk)	CATAGAATATTCGAGCTCCACCGCGGTG
	HR-R	4	Pv pyrG 3'flk F(MCS)	CGGTGGAGCTCGAATATTCTATGATCTGTCTTTTTATATTTG
			Pv pyrG 3'flk R(ApaI)	GATGCATGGGCCCGACACTCTCCACGAG
SP-eGFP	signal seq	5	Pv tanSP F(NotI)	GAATGCGGCCGCATCCACTCGTTCACTTG
			Pv tanSP R(eGFP)	CTTGCTCACCATTGCATTGGCGGACGCTG
	eGFP	6	eGFP F(Pv tanSP)	CCGCCAATGCAATGGTGAGCAAGGGCGAG
			eGFP R(NotI)	GAATGCGGCCGCAGGGTATTGCCTTTGGC
Promoters	P_{PH4}	7	Pv PH4 F(SalI)	GTCGACAGATGAAGGCGTTGATAG
			Pv PH4 R(SpeI)	ACTAGTCTGCTTGGTTCTAGCC
	P_{P2}	8	Pv PP2 F(SalI)	GTCGACGGAGCTTAAGAACTGC
			Pv PP2 R(SpeI)	ACTAGTAAGCGGCGAGGTGCTTC
	P_{S8}	9	Pv PS8 F(SalI)	GTCGACGCGATCGTGAGAAAGG
			Pv PS8 R(SpeI)	ACTAGTAGAAGGCCCTCTTCGC
	P_{tub}	10	Pv Pbt F(SalI)	GTCGACAGTACTGGGGCATCG
			Pv Pbt R(SpeI)	ACTAGTCAACAAACGTACGATTTCAC
	P_{tef1}	11	Pv Ptef1 F(SalI)	GTCGACCACAGCTACGTATCG
			Pv Ptef1 R(SpeI)	ACTAGTCCGAATGAAGCCCATC
	P_{gpdA}	12	Pv PgpdA F(SalI)	GTCGACCTAGAAATTCCGGCTAAAG
			Pv PgpdA R(SpeI)	ACTAGTGGATTCGCATCAACAAC
P_{gpdA}-eGFP	P_{gpdA}	13	Pv PgpdA F(SalI)	GTCGACCTAGAAATTCCGGCTAAAG
			Pv PgpdA R(eGFP)	CTTGCTCACCATTTTGTTTGAATTGACACCGGC
	eGFP	14	eGFP F(Pv PgpdA)	ATTCAAACAAAATGGTGAGCAAGGGCGAG
			eGFP R(NotI)	GAATGCGGCCGCAGGGTATTGCCTTTGGC
pyrG		15	Pv pyrG flk F	CAGCTTCCAGACCTTCAGG
			Pv pyrG flk R	CGACACTCTCCACGAGTC
Reverse transcription	tubulin	16	Pv bt RT F	GCTGGCAAGAAGTATGTTCC
			Pv bt RT R	CAGCTGGTGAACGGAGAG
	eGFP	17	Pv eGFP RT F	CGACGTAAACGGCCACAAG
			Pv eGFP RT R	CTTCTGCTTGTCGGCCATG
